# Delineating Tissue‐Specific Cell Identity of Oral Mucosa in Humans and Mice From a Single‐Cell Perspective

**DOI:** 10.1111/jcmm.70768

**Published:** 2025-08-01

**Authors:** Huanyu Luo, Xiaoyi Yu, Hongchen Sun, Zhengwen An

**Affiliations:** ^1^ Department of Oral Biology, School and Hospital of Stomatology Jilin University Changchun China; ^2^ Key Laboratory of Tooth Development and Bone Remodeling of Jilin Province, School and Hospital of Stomatology Jilin University Changchun China; ^3^ Department of Oral Pathology, School and Hospital of Stomatology Jilin University Changchun China

**Keywords:** fibroblasts, oral mucosa, single‐cell transcriptomics, translation medicine, wound healing

## Abstract

The oral mucosa exhibits superior healing and minimal scarring. Although mouse models are widely used to study wound healing and various diseases, their translational relevance remains unclear. Here, we performed a comparative single‐cell transcriptomic analysis of human and mouse oral mucosa to identify both shared and species‐specific mechanisms. A total of 34,969 cells from human and mouse datasets were integrated using Harmony for batch effect correction, allowing us to establish a unified oral mucosa transcriptome atlas. Fibroblasts emerged as the prominent cell population in both species, displaying conserved gene expression profiles and cell communication networks, underscoring their central role in tissue homeostasis. Key pathways involved in extracellular matrix remodelling and wound healing were highly conserved, supporting the utility of mouse models for studying fibroblast‐mediated tissue regeneration. These findings suggest that mouse models can effectively replicate human fibroblast biology, offering valuable insights for developing translational therapies that target fibroblast activity and regulatory gene networks to enhance wound healing and tissue regeneration. Additionally, we identified species‐specific cell populations, including human‐specific capillary endothelial cells and melanocytes, as well as mouse‐specific salivary gland epithelial cells. Their distinct cellular composition and functional differences suggest that these subpopulations may not be directly translatable from mouse models to human contexts. Overall, our study highlights the evolutionary conservation of fibroblasts while identifying species‐specific differences that warrant consideration in translational research. These findings provide a valuable resource for researchers using mouse models to study oral mucosa‐related diseases, facilitating the translation of preclinical discoveries into clinical applications.

## 1 Introduction

The oral mucosa served as an ideal model for wound healing research [1, 2, 3] and has been extensively used to investigate the molecular mechanisms underlying its superior healing capacity compared to the skin [3, 4, 5]. Although mice are widely used models, the species‐specific differences may limit the direct translation of these findings to human wound healing. For example, a comparative study showed that mice and humans share common cell types in certain tissues; the relative proportions and functional characteristics of these cells can differ significantly [6]. Studies have shown that the immune compartment of adipose tissue and macrophage populations vary between humans and mice [7, 8]. Additionally, tissue‐specific gene expression, such as ATPase inhibitor 1 [9] and selenoprotein, displays significant interspecies differences, which may influence physiological functions [10]. These variations underscore the necessity of carefully interpreting mouse model data when translating findings to human biology.

Advances in single‐cell RNA sequencing (scRNA‐seq) have provided valuable insights into cellular diversity and molecular signalling across species [11]. For instance, Drake et al. identified a unique subpopulation of stromal cells with inflammatory signatures in human mucosa, which is linked to neutrophil recruitment, uncovering the cell‐specific expression patterns of periodontitis susceptibility genes by scRNA‐seq [12]. Furthermore, Liu et al. identified a previously unrecognised immune cell landscape associated with periodontitis and colitis progression at the single‐cell level, offering new insights into strategies for controlling and preventing inflammatory bowel disease (IBD) exacerbations [13]. Kang et al. used scRNA‐seq and lineage tracing to identify *Prx1* ^+^ fibroblasts as a key subpopulation that accelerates mucosal healing by enhancing early immune responses. Notably, comparative analyses have identified *Prx1* ^+^ progenitors in human oral mucosa that exhibit spatial and transcriptional similarities to their murine counterparts. These progenitors demonstrate conserved differentiation trajectories and cellular communication networks, suggesting that targeting similar stromal subsets in humans could inform novel therapeutic strategies for optimising wound regeneration [14]. These findings underscore the importance of cross‐species comparisons in advancing our understanding of oral mucosal biology and its implications for wound healing and disease.

While mouse models remain widely used, alternative models such as porcine systems and 3D in vitro organoid cultures provide complementary approaches for studying wound healing and regeneration. Murine models offer robust genetic tools and established transgenic lines, making them ideal for dissecting molecular mechanisms. However, due to their small size and inherent biological differences, they do not fully recapitulate the structural and physiological features of human skin and oral mucosa. In contrast, porcine skin closely resembles human skin in both structure and function [15, 16]. For instance, pigs and humans share a similar epidermal thickness and dermal/epidermal ratio, along with well‐developed rete ridges and dermal papillae [17, 18]. Importantly, pigs lack the panniculus carnosus, a subdermal muscle layer present in rodents that promotes rapid wound contraction, resulting in a wound healing pattern that more closely mimics the re‐epithelialisation process seen in humans [19]. Additionally, porcine dermis exhibits comparable vascular architecture, epidermal turnover time, keratin expression and lipid composition of the stratum corneum [20]. These characteristics make porcine skin a highly translational model for studying human wound healing.

The integration of porcine and mouse models with in vitro organoid systems allows for multi‐level analysis of wound healing mechanisms, from molecular signalling to tissue‐level responses. Such a multi‐model approach, supported by previous studies [21, 22], enhances the robustness and clinical relevance of experimental findings, facilitating the development of more effective regenerative therapies. Despite these advances, a comprehensive comparative analysis of the cellular landscape of human and mouse oral mucosa remains limited. This study aims to systematically delineate the similarities and differences between human and mouse oral mucosa at a single‐cell resolution. By integrating scRNA‐seq approaches, we seek to provide a refined framework for understanding how findings from mouse models can be effectively translated to human biology. This knowledge will enhance the application of mouse experimental models in wound healing research, facilitate clinical translation and inform therapeutic strategies for accelerating human skin repair.

## 2 Materials and Methods

### 2.1 Animal Work

All animal work was carried out under the guidelines of the Laboratory Animal Ethics Committee, School of Basic Medical Sciences, Jilin University, China, in compliance with the Declaration of Helsinki (Approval no. 2023493). This study complied with the protocol under updated ARRIVE 2.0 guidelines.

C57BL/6 mice were purchased from Beijing Vital River Laboratory Animal Technology Co. Ltd. They were housed in a specific pathogen‐free (SPF) environment, provided unrestricted access to food and water, maintained at a stable temperature of 22°C and 55% ± 10% humidity, under a consistent 12‐h light–dark cycle.

### 2.2 Single‐Cell RNA Sequencing on Mouse Oral Mucosa

Oral mucosa samples were collected from five healthy 8‐week‐old mice. Single‐cell suspensions of the mouse oral mucosa were obtained through enzymatic digestion. Cell quality was initially assessed based on the following criteria: viability > 85%, a cell concentration of approximately 1000 cells/μL, and a cell aggregation rate < 15%, meeting the requirements for single‐cell RNA sequencing. A total of 10,000 cells were targeted and loaded onto a 10X Chromium system, using the Chromium Single‐Cell v3.1 reagent kit, following the manufacturer's instructions. Sequencing was performed on the NextSeq 500 platform (Illumina) with paired‐end sequencing and dual indexing. Raw sequencing data were generated in FASTQ format and processed using the Cell Ranger pipeline (10X Genomics).

Further cell and gene quality control was performed using the Seurat R package. Low‐quality cells and genes with abnormal expression were filtered out. Specifically, cells were excluded if they expressed fewer than 200 genes (min.features = 200), and genes expressed in fewer than 3 cells (min.cells = 3) were also removed. Empty GEMs and GEMs containing multiple cells (multiplets) were excluded. High‐quality cells were retained based on the following thresholds: nFeature_RNA ≥ 250; nCount_RNA > 1000; percent.mt < 20%. To ensure transcriptomic complexity, only cells with a log10(GenesPerUMI) > 0.8, calculated as log10(nFeature_RNA)/log10(nCount_RNA), were included. Doublets were further identified and removed computationally. Only cells that passed all quality control criteria were retained for downstream analysis.

### 2.3 Collection of Publicly Available Datasets on Human Oral Mucosa

Single‐cell RNA sequence data of human buccal mucosa were downloaded from the GSE164241 (https://www.ncbi.nlm.nih.gov/geo/query/acc.cgi?acc=GSE164241). Samples with over 3000 cells after data processing (BM157, BM158, BM168, BM169) were retained for further analysis.

### 2.4 Human and Mouse scRNA‐Seq Data Integration

Seurat was selected for its robust and widely validated framework for single‐cell RNA sequencing (scRNA‐seq) analysis. It provides comprehensive preprocessing, normalisation, integration and clustering methods, making it particularly effective for handling batch effects and cross‐species comparisons. Additionally, Seurat's ability to perform dimensionality reduction and visualisation (e.g., UMAP) enables a clear and interpretable representation of cellular heterogeneity.

Gene expression matrices were extracted from human mucosal tissues scRNA‐seq datasets, and gene symbols from mouse were mapped to human. The ‘SCTransform’ function from the Seurat package was applied to normalise the merged gene expression data, correcting for technical noise while preserving biological variability. ‘Harmony’ algorithm was then employed to perform batch effect correction, removing unwanted technical variation between different batches.

### 2.5 Data Dimensionality Reduction and Unsupervised Clustering

The Seurat package's ‘FindVariableFeatures’ function was used to obtain highly variable genes (HVGs) as feature genes. Principal Component Analysis (PCA) was employed on merged data through ‘RunPCA’ function in Seurat. Significant principal components (PCs) were selected based on the elbow plot of variance. Cells were clustered using the ‘FindNeighbors’ (*k* = 20) and ‘FindCluster’ (resolution = 0.3) functions, employing a graph‐based Louvain algorithm. Uniform Manifold Approximation and Projection (UMAP) was applied for further visualisation in 2D or 3D space.

### 2.6 Pearson Correlation Analysis

The SCpubr package's ‘do_CorrelationPlot’ function was used to perform Spearman correlation analysis of annotated cell type populations between human and mouse integrated mucosa datasets. The coefficient quantifies the degree of linear relationship between two variables, ranging from −1 (perfect negative correlation, blue) to 1 (perfect positive correlation, red), with 0 indicating no correlation (white). Pearson's correlation was assessed with a significance level (*α*) of 0.05. A typical confidence level of 95% was used for the correlation coefficients.

### 2.7 CellChat Analysis

CellChat was chosen to analyse intercellular communication by inferring ligand‐receptor interactions from scRNA‐seq data. Additionally, the database of CellChat integrates curated ligand‐receptor interactions across multiple species, making it particularly suitable for our cross‐species analysis.

The CellChat package from the CellChatDB database was employed, incorporating prior knowledge of signal transduction and gene regulatory networks. A probability value was calculated based on the average expression values of ligands, receptors and their cofactors. The probability of cell interactions was recalculated through statistical tests to determine significant interactions. Wilcoxon rank‐sum test with the significance level of 0.05 was used for the statistical test to recalculate the probability of cell interactions.

### 2.8 Enrichment Analysis

Enrichment analysis results were obtained using the g:Profiler web tool (https://biit.cs.ut.ee/gprofiler/). Results from Gene Ontology, KEGG, Reactome and WikiPathways databases were visualised using the ‘ggplot2’ package.

### 2.9 Differentially Expressed Genes (DEGs) and Cell Type Annotation

DEGs were identified using the ‘FindAllMarkers’ function in the Seurat package with the Wilcoxon rank‐sum test (adjusted *p*‐value < 0.05, logfc.threshold = 0.5). Only genes with a log fold‐change greater than 0.5 and an adjusted *p*‐value less than 0.05 were retained. Conserved genes were defined as the intersection of DEGs identified in the same cluster of human and mouse mucosa. Clusters were annotated using canonical marker genes (e.g., EPCAM for epithelial cells, PTPRC for immune cells).

### 2.10 Cell Trajectory Analysis

ScRNA‐seq data of fibroblast clusters were preprocessed and normalised, then converted into a cell data set (CDS) for pseudotime analysis. The ‘Monocle3’ package was used to learn the differential trajectory and depict pseudotime. Differentially expressed genes along the pseudotime trajectory were identified and analysed.

## 3 Results

### 3.1 Single Cell Landscape of Oral Mucosa in Humans and Mice

**FIGURE 1:**
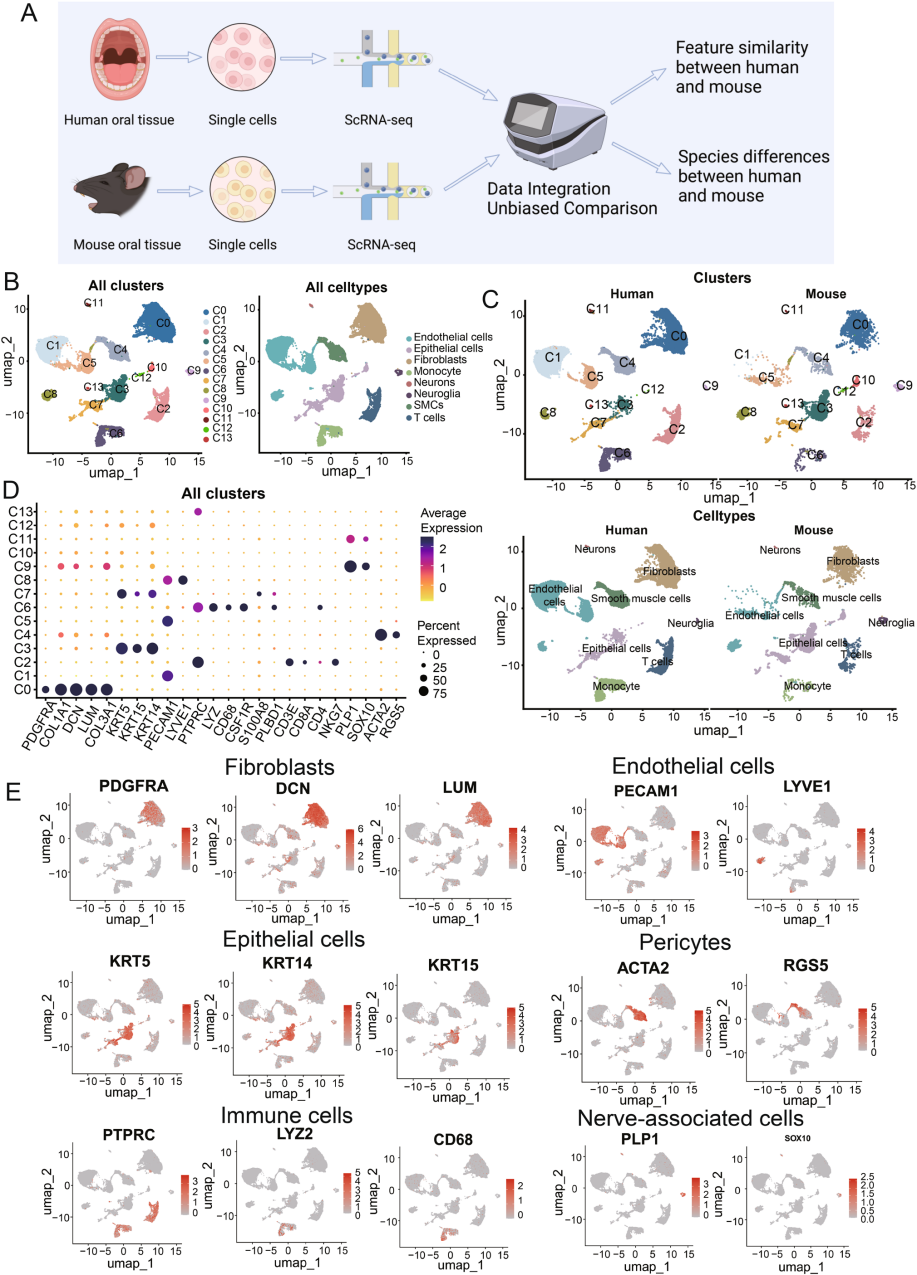
Single cell landscape of oral mucosa in humans and mice. (A) Flowchart for the comparative analysis of single‐cell data from human and mouse oral mucosa. Harmony is used for batch effect correction. (B) Manifold projection of all scRNA‐seq cells, coloured by cell type. (C) Manifold projection of scRNA‐seq cells of human and mouse oral mucosa, coloured by clusters and cell types, respectively. (D) Dot plot showing the expression of representative genes for each cluster. The node size positively correlates with the percentage of a given cell type of positive for a given marker. The colour key, from light yellow to dark blue, indicates low to high gene expression levels. (E) UMAP plots highlighting the representative genes of each cell type. The colour key, from grey to red, indicates low to high gene expression levels.

To gain the systematical overview of the similarities and disparities between mouse and human oral mucosa, we performed single‐cell sequencing on healthy mouse oral mucosa and integrated the dataset with previously published scRNA‐seq data of human oral mucosa. A total of 34,969 cells were included in the study, comprising four groups of single‐cell datasets from human samples and one group of single‐cell data from our own dataset on mouse oral mucosa (Figure 1A). To integrate the datasets from both species, we first converted mouse genes to human homologues. Subsequently, the Harmony algorithm was applied for batch effect correction (Figure S1). The uniform manifold approximation and projection (UMAP) dimension reduction effectively integrates the human and mouse datasets, revealing the heterogeneity of cells with similar identities rather than completely different datasets (Figure 1B,C). We obtained a total of 14 cell groups through dimensionality reduction clustering, including fibroblasts (PDGFRA, DCN, LUM), epithelial cells (KRT5, KRT14, KRT15), endothelial cells (PECAM1, LYVE1), immune cells (PTPRC), pericytes (ACTA2, RGS5) and cells associated with the nervous system (SOX10, PLP1, MPZ) (Figure 1C,D). The representative gene markers were highlighted on UMAP showing each cell type, respectively (Figure 1E). Taken together, by aggregating recently published, publicly available oral mucosal transcriptomic datasets of humans and integrating them with our own datasets from mice, we established a comprehensive transcriptome atlas of oral mucosa, capturing its cellular diversity. This atlas normalises the single‐cell dataset of human and mouse oral mucosa, allowing us to perform an unbiased interspecies comparative analysis of these cell types.

### 3.2 Comparative Analysis of Cellular Profiles Between Human and Mouse Oral Mucosa

**FIGURE 2:**
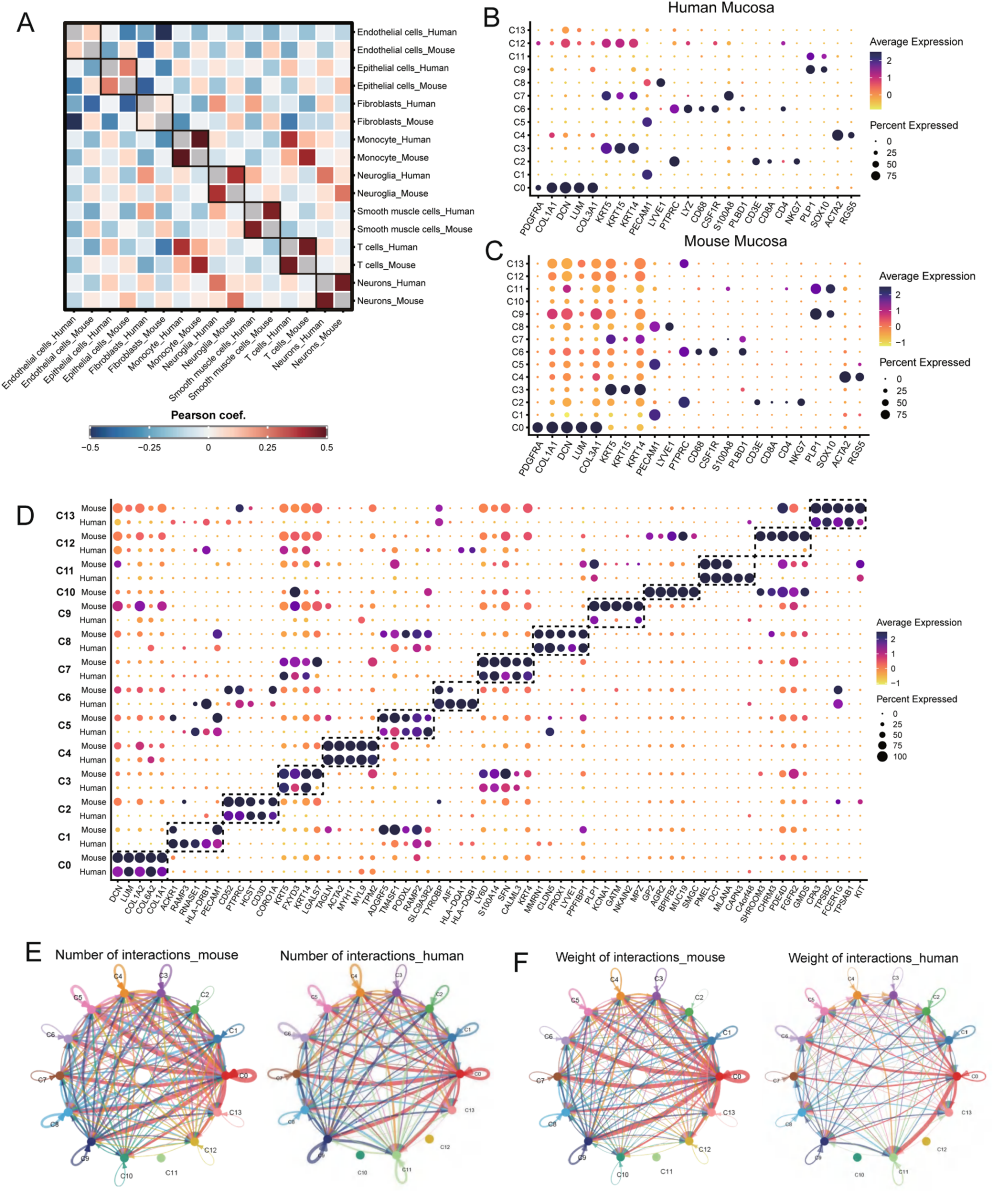
Comparative analysis of cellular profiles between human and mouse oral mucosa. (A) Pearson correlation coefficients comparing cell types across human and mouse oral mucosa. Pearson's correlation was assessed with a significance level (*α*) of 0.05. A typical confidence level of 95% was used for the correlation coefficients. (B, C) Dot plots showing the expression of representative genes for each cell type in human and mouse oral mucosa, respectively. The node size positively correlates with the percentage of a given type of cells positive for a given marker. The colour key, from light yellow to dark blue indicates low to high gene expression levels. (D) Dot plot showing the expression of the top shared common genes in each cell population across human and mouse oral mucosa. The node size positively correlates with the percentage of a given type of cells positive for a given marker. The colour key, from light yellow to dark blue, indicates low to high gene expression levels. (E, F) Circos plots depicting putative ligand‐receptor interactions among all clusters in human and mouse oral mucosa reveal that C0 exhibits the strongest interactions in both species. The edge width represents the number (E) and the weight (F) of interactions in each cell group. Different colours and numbers represent different cell clusters.

We initially assessed the correlation of overall cell types between human and mouse oral mucosa using single‐cell datasets and Pearson's correlation coefficient analysis. The results revealed a positive correlation for each cell type, indicating general comparability between the two species (Figure 2A). By analysing the top marker genes in each cell cluster, we observed a very similar gene distribution pattern in both humans and mice, as visualised by dot plots (Figure 2B,C). We then combined the two datasets to identify common genes in each cell population across the two species. Despite some differences in gene expression, the genes expressed as classical markers defining each cell subset were largely consistent between humans and mice (Figure 2D).

Additionally, we used CellChat analysis to evaluate the communication strength between different cell subsets in human and mouse oral mucosa (Figure 2E,F and Figure S2). We compared both the variation in numbers (Figure 2E) and the intensity (Figure 2F) of cell communication. Our findings demonstrated general similarity between the two datasets. Notably, the signal interactions of the C0, a fibroblast subset, were the most significant in both number and strength, highlighting the importance of the fibroblast in tissue homeostasis across species. Analysis of outgoing signalling patterns revealed that C0 exhibited the strongest signals in both human and mouse, particularly collagen [23, 24] and laminin signals [25, 26] signalling pathways, which are essential for maintaining tissue homeostasis and promoting wound healing. Additionally, FN1, tenascin and Thbs signalling pathways [27, 28, 29, 30, 31] were identified as key contributors to tissue repair and wound healing. Overall, there are fundamental conservations in marker gene expression and cell‐to‐cell interactions among different cell populations in human and mouse oral mucosa. This indicates that studying mouse oral mucosa can provide valuable insights applicable to human research.

### 3.3 Fibroblast Consistency and Their Importance in Tissue Homeostasis

**FIGURE 3:**
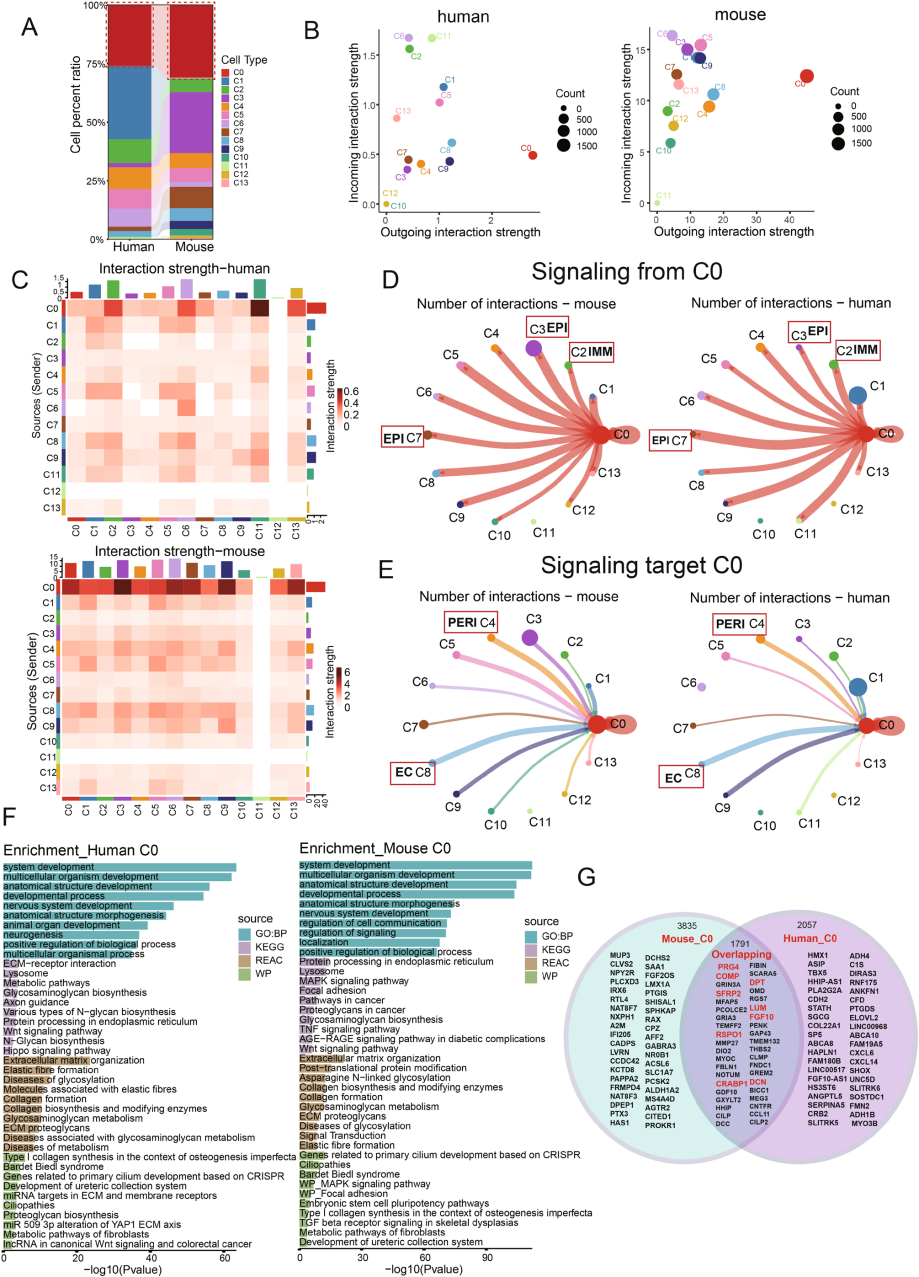
Fibroblast consistency and their role in tissue homeostasis. (A) Histogram showing the similar proportion of fibroblasts in human and mouse oral mucosa. (B) Volcano plot illustrating the incoming and outgoing interaction strength of all cell types, with the C0 fibroblasts apart from all other cell type displaying the unique strong interaction from both human and mouse oral mucosa. (C) CellChat analysis of interaction strength across all cell types, revealing the strongest interactions in fibroblasts in both human and mouse oral mucosa. (D, E) Circos plots displaying similar putative ligand‐receptor interactions between fibroblasts (C0) and other cell clusters in human and mouse oral mucosa, respectively. The edge width represents the number of interactions from (D) and target fibroblasts (E). Different colours and numbers correspond to different cell clusters. (F) Functional enrichment analysisusing GO, KEGG, WIKI and REACTOME databases, highlighting similar pathways in fibroblasts from human and mouse oral mucosa. (G) Venn diagram showing the shared and distinct highly expressed markers between human and mouse oral mucosal fibroblasts.

We examined the cell proportions and found that fibroblasts are the major cell type in both human and mouse tissues, with similar proportions in both species (Figure 3A). By analysing the outgoing and incoming interaction strengths in humans and mice, we identified C0 fibroblasts as the most prominent cell population, distinguishing themselves from all other cell populations (Figure 3B). Using interaction strength analysis, we found that C0 fibroblasts displayed the strongest interactions with all other cell clusters both in human and mouse (Figure 3C), indicating their dominant function in the oral mucosa. We next examined the outgoing signals from C0 fibroblasts and the incoming signals targeting C0 fibroblasts, identifying a similar pattern in both directions (Figure 3D,E). This suggests a conserved network of fibroblast interactions with other niche cells in the oral mucosa between humans and mice. Gene enrichment analysis of this fibroblast cluster revealed shared biological functions such as ‘system development’, ‘multicellular organism development’ and ‘developmental process’, underscoring their potential role in tissue homeostasis and regeneration. Pathway enrichment analysis further demonstrated that Wnt signalling was among the top 10 enriched pathways in both human and mouse fibroblasts in the KEGG database (Figure 3F), highlighting its crucial role in fibroblast function and its evolutionary conservation across species. This aligns with previous studies showing that the Wnt signalling is conserved pathway involved in tissue repair, including fibroblast recruitment, proliferation and collagen deposition [32, 33].

Comparing the genes expressed in oral mucosa fibroblasts, we identified a total of 1791 co‐expressed genes in both humans and mice. The Venn diagram showed the top 20 genes shared by human and mouse fibroblasts (Figure 3G), which include well‐recognised classic fibroblast markers such as LUM and DCN, as well as a number of shared genes that have been well studied in human fibroblasts, such as CRABP1, SFRP2, PRG4, RSPO1, COMP and DPT. CRABP1‐mediated RA signalling has been widely studied in tissue regeneration [34, 35], and SFRP2 and PRG4 have defined distinct subtypes of human fibroblasts that play roles in matrix deposition, inflammatory cell retention and connective tissue cell differentiation [36]. COMP has also been shown to enhance DNA synthesis and cell cycle progression in human periodontal ligament cells through Tie2‐mediated phosphorylation of PI3K/Akt and MAPK [37].

In conclusion, fibroblasts share similar basic characteristics and functions in humans and mice, making them valuable research subjects in studies using mice as an experimental model. These common fibroblast‐enriched genetic markers that we identified may be useful references in future animal studies and applied translation.

### 3.4 A Distinct Fibroblast Subset With Stem Cell Characteristics in Both Humans and Mice

**FIGURE 4:**
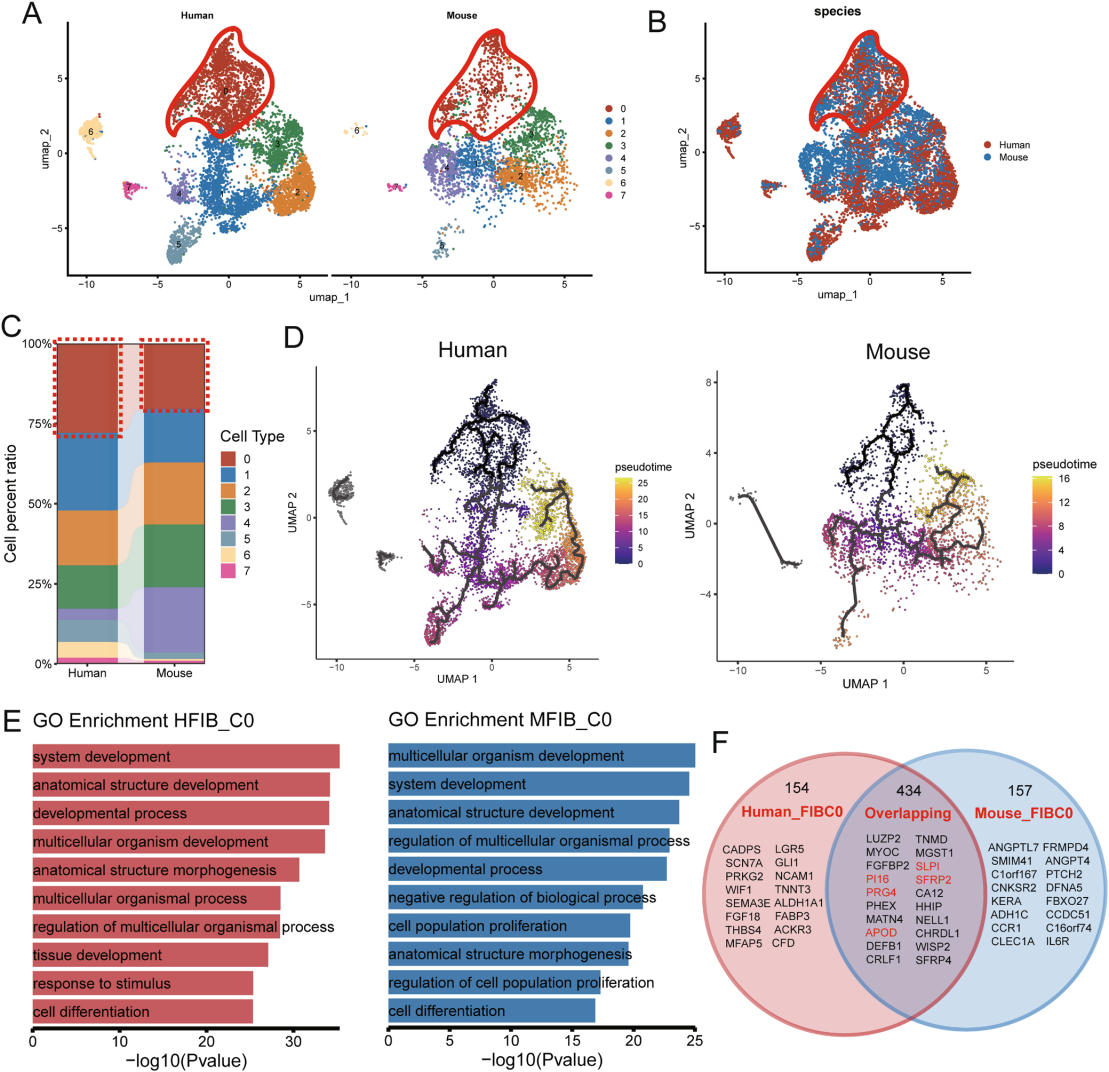
A distinct fibroblast subset with stem cell characteristics in both humans and mice. (A) Manifold projection of fibroblast subsets in human and mouse oral mucosa, showing the similar distribution of FIB C0 subset in both species, coloured by cell type. (B) Manifold projection demonstrating excellent overlap of FIB C0 fibroblast subsets in human and mouse oral mucosa, coloured by species. (C) Histogram showing the proportion of all fibroblast subsets, highlighting the similarity of the fibroblast subset C0 (FIB C0) between human and mouse oral mucosa. (D) Monocle 3 trajectory analysis revealing the putative differentiation trajectory of fibroblast subsets from human and mouse oral mucosa, indicating that FIB C0 cells are at the initial differentiation point and poised to mature into various fibroblast subsets. (E) GO enrichment analysis of fibroblast subset C0 (FIB C0) from human and mouse oral mucosa, highlighting the enrichment of development and differentiation pathways. (F) Venn diagram displaying the shared and distinct highly expressed markers between human and mouse oral mucosal fibroblast subset FIB C0.

To gain deeper insights into fibroblasts heterogeneity in the oral mucosa, we subdivided all fibroblasts into eight sub‐populations. UMAP analysis revealed a similar distribution of these fibroblast subpopulations in both human and mouse tissues (Figure 4A). Each cluster showed overlapping distributions (Figure 4B) and comparable proportions (Figure 4C), with subcluster C0 fibroblasts (FIB C0) displaying the strongest cross‐species correspondence (Figure 4B). Using the Monocle 3 algorithm, we reconstructed a differentiation trajectory to explore fibroblast fate decisions. Our analysis identified FIB C0 as positioned at the origin of the differentiation trajectory, suggesting its role as undifferentiated progenitor‐like population in both humans and mice oral mucosa (Figure 4D). GO enrichment analysis further supported this, highlighting conserved biological processes such as ‘system development’, ‘anatomical structure development’, ‘developmental process’ and ‘multicellular organism development’ in both species (Figure 4E). These findings reinforce the stem‐like properties of this subpopulation and their potential role in maintaining oral mucosa homeostasis.

To investigate cross‐species conservation, we compared the gene expression profiles of human and mouse FIB C0 subpopulations using differential gene analysis. The results revealed a significant overlap in gene expression between the two species, including classical fibroblast markers such as DCN and LUM, as well as genes involved in matrix formation and wound healing, such as PRG4, THBS2 and SLPI [38, 39, 40]. Notably, both species shared several genes associated with stem cell functions, including PI16, SFRP2 and APOD [39, 41, 42, 43, 44] (Figure 4F).

Given their conserved stem‐like properties, FIB C0 fibroblasts may serve as key regulators of wound healing across different tissue environments. Their ability to secrete extracellular matrix components and modulate immune responses suggests they could contribute to tissue regeneration by maintaining a pro‐repair niche. In oral mucosa, where healing is rapid and scar‐free, these fibroblasts may promote a regenerative microenvironment through enhanced ECM remodelling and immune crosstalk. In contrast, in other tissues with slower healing dynamics, such as skin, their role may be more constrained by local inflammatory and fibrotic signalling. Understanding how these fibroblasts adapt to distinct tissue environments could provide novel therapeutic strategies for enhancing wound healing and reducing fibrosis in chronic wounds.

### 3.5 Human Tissue‐Distinctive Cell Subsets in Oral Mucosa

**FIGURE 5:**
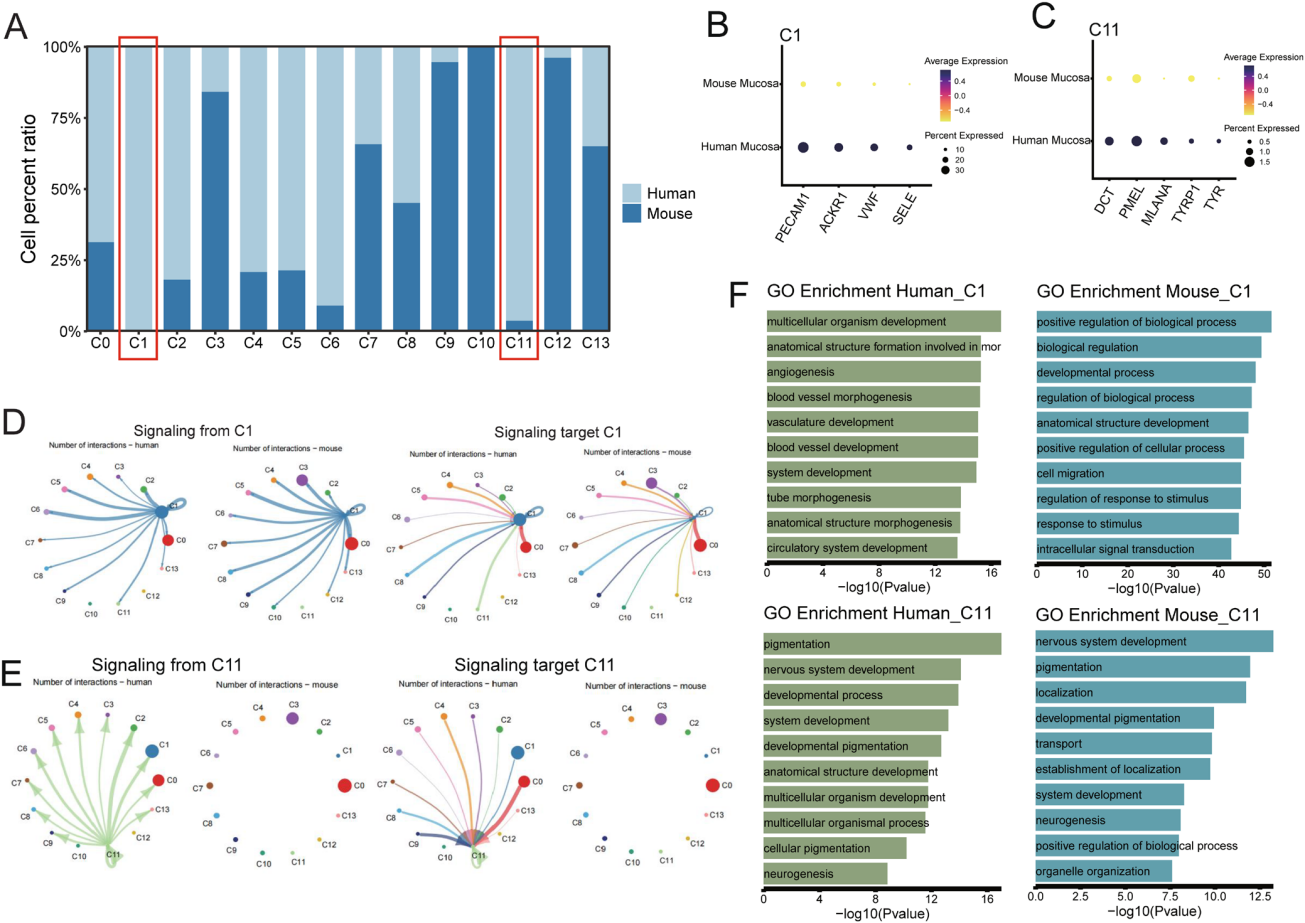
Human tissue‐distinctive cell subsets in oral mucosa. (A) Histograms showing the relative proportion differences of the various cell types between human and mouse oral mucosa, indicating that C1 and C11 are predominantly found in human, but not in mouse oral mucosa (no statistical tests were applied). (B, C) Dot plot depicting the different genes expression of C1 (B) and C11 (C) in human and mouse oral mucosa, respectively, revealing that classical markers of C1 and C11 are specifically expressed in humans. The node size positively correlates with the percentage of a given type of cells positive for a given marker. The colour key, from light yellow to dark blue, indicates low to high gene expression levels. (D, E) Circos plots displaying putative ligand‐receptor interactions between C1 (D), C11 (E) and other cell clusters from human and mouse oral mucosa, respectively. These plots highlight that C1 interactions are similar in both species, whereas C11 interactions are primarily observed in humans. Edge width represents the number of interactions in the left (source) and right (target) panels for C1 and C11, respectively. Different colours and numbers represent different cell clusters. (F) GO enrichment analysis showing similar pathways enriched in C1 and C11 from human and mouse oral mucosa, suggesting that these subsets share similar physiological functions.

Despite the overall similarities between human and mouse oral mucosa, our single‐cell atlas reveals distinct differences in specific cell populations. By comparing the proportions of each cell cluster, we identified two human‐exclusive clusters: C1 (post‐capillary endothelial cells) and C11 (melanogenic cells) (Figure 5A and Figure S3). Cluster C1 expresses ACKR1, VWF, PLVAP, SELE and RAMP3, which are hallmarks of post‐capillary endothelial cells [45, 46, 47, 48] (Figure 5B). These cells constitute approximately 31% of the total human oral mucosal cells, compared to only 0.28% in mice, indicating a significantly enriched capillary endothelial network in humans. GO enrichment analysis revealed that while post‐capillary endothelial cells share conserved functions in angiogenesis, vasculature development and blood vessel morphogenesis across species, human C1 cells exhibit a more prominent role in vascular permeability regulation and inflammatory response (Figure 5F). Given the functional significance of post‐capillary endothelial cells in material exchange, immune surveillance and tissue repair [49, 50], their scarcity in the mouse oral mucosa may impact microvascular‐related processes. This discrepancy underscores a key limitation of using mouse models for studying oral mucosal microcirculation and vascular‐related pathologies, necessitating alternative models, such as porcine or human‐derived organoid systems, for translational validation.

Cluster C11 comprises melanogenic cells, characterised by the expression of DCT, PMEL, MLANA, TYRP1 and TYR [51, 52] (Figure 5C). Unlike humans, where melanocytes are present in both skin and oral mucosa [53, 54, 55], mice lack melanogenic cells in their oral mucosa. GO enrichment analysis revealed that pigmentation and nervous system development are the predominant biological processes in both species (Figure 5F). However, beyond mere presence/absence, our signalling interaction analysis showed that C11 cells engage in significantly more intercellular interactions in human tissues compared to mice (Figure 5E), suggesting a more integrated role in local tissue homeostasis. This could have functional implications, particularly in melanin‐related immune modulation and barrier protection in the human oral mucosa. The absence of melanocytes in mice suggests that they may be less suitable for studying oral mucosal pigmentation disorders and melanocyte‐associated diseases, such as oral mucosal melanoma.

Taken together, species‐specific differences in the composition, abundance and functional integration of oral mucosal cell populations must be carefully considered when extrapolating findings from mouse models to human biology. While mice remain a valuable model for studying general oral mucosal processes, the absence of capillary endothelial cells and melanocytes presents limitations in vascular, inflammatory and pigmentation‐related research, necessitating the use of alternative models that better mimic human physiology.

### 3.6 Mouse Tissue‐Prevalent Cell Subsets in Oral Mucosa

**FIGURE 6:**
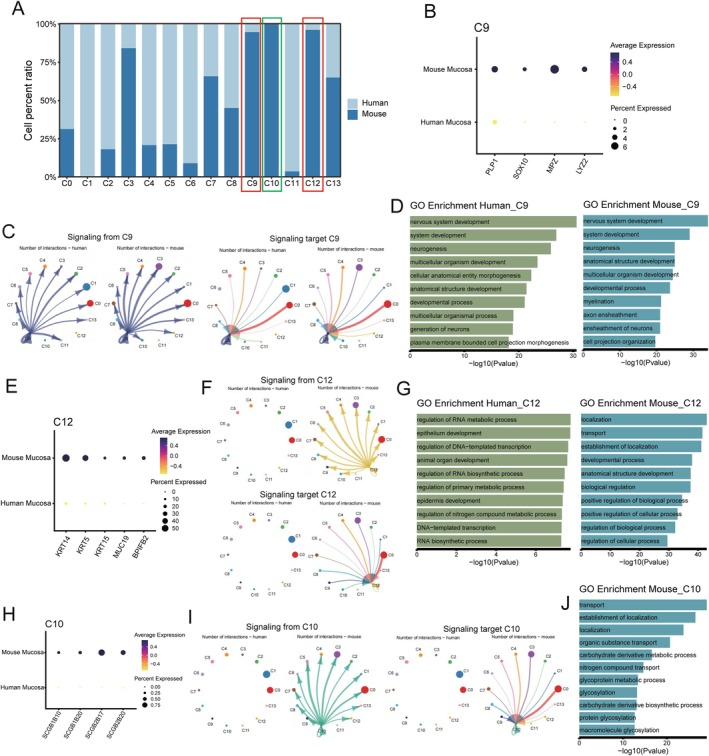
Mouse tissue‐prevalent cell subsets in oral mucosa. (A) Histograms showing the relative proportion differences of the various cell types between human and mouse oral mucosa, highlighting that C9, C10 and C12 are predominantly found in mouse, but not in human, oral mucosa (no statistical tests were applied). (B) Dot plot showing the different genes expression of C9 in human and mouse oral mucosa, revealing that classical markers of C9 are specifically expressed in mice. The node size positively correlates with the percentage of a given type of cells positive for a given marker. The colour key, from light yellow to dark blue, indicates low to high gene expression levels. (C) Circos plots displaying putative ligand‐receptor interactions between C9 and other cell clusters from human and mouse oral mucosa. Edge width represents the number of interactions from (left panel) and target (right panel) C9. Different colours and numbers represent different cell clusters. (D) GO enrichment analysis showing signals enriched with C9 from human and mouse oral mucosa, suggesting that C9 shares similar physiological functions across species. (E) Dot plot showing the differential gene expression of C12 in human and mouse oral mucosa, revealing that classical markers of C12 are specifically expressed in mice. The node size positively correlates with the percentage of a given type of cells positive for a given marker. The colour key, from light yellow to dark blue, indicates low to high gene expression levels. (F) Circos plots displaying putative ligand‐receptor interactions between C12 and other cell clusters, showing that C12 interactions are mainly observed in mouse oral mucosa but not in human. Edge width represents the number of interactions from (upper panel) and target (lower panel) C12. Different colours and numbers represent different cell clusters. (G) GO enrichment analysis showing signals enriched with C12 from human and mouse oral mucosa, suggesting that C12 shares similar physiological functions across species. (H) Dot plot showing the differential gene expression of C10 in human and mouse oral mucosa, revealing that classical markers of C10 are specifically expressed in mouse oral mucosa, but not in human. The node size positively correlates with the percentage of a given type of cells positive for a given marker. The colour key, from light yellow to dark blue, indicates low to high gene expression levels. (I) Circos plots displaying putative ligand‐receptor interactions between C10 and other cell clusters, showing that C10 interactions are primarily observed in mouse oral mucosa, but not in human. Edge width represents the number of interactions from and target C10. Different colours and numbers represent different cell clusters. (J) GO enrichment analysis showing signals enriched in C10 from mouse oral mucosa, highlighting the species specificity of C10 in mouse oral mucosa.

We identified several cell subpopulations that are predominant in mouse oral mucosal tissues, including C9, C10 and C12 (Figure 6A and Figure S3). Among these, C9 represents a neural glial cell population characterised by the expression of classical markers such as PLP1 and SOX10 (Figure 6B). Peripheral nerves are critical for tissue homeostasis and repair, as they not only regenerate rapidly following injury but also establish direct interactions with fibroblasts, keratinocytes and endothelial cells to modulate wound healing [56, 57, 58]. Although the CellChat interaction analysis (Figure 6C) and GO enrichment analysis (Figure 6D) revealed functional similarities between mouse and human C9 subsets, the significantly higher abundance of neural glial cells in the mouse oral mucosa suggests a more pronounced role in nerve‐associated tissue repair. These cells may contribute to enhanced neurogenic signalling, immune modulation and extracellular matrix remodelling, all of which influence the wound healing microenvironment. Given their scarcity in human oral mucosa, this species‐specific difference highlights a potential limitation of using mouse models to study neural glial cell function in human oral tissue regeneration and neuropathic conditions. Understanding how these cells interact with other stromal and immune components could provide valuable insights into species‐specific mechanisms of wound healing and sensory regulation. Cluster 12, a subpopulation of epithelial cells, expresses classical epithelial markers, including KRT14, KRT5 and KRT15, along with salivary mucin‐associated genes such as MUC19 [59, 60] and BPIFB2 [61] (Figure 6E). This indicates their association with salivary gland function. Notably, CellChat and GO enrichment analyses revealed functional differences between mouse and human C12 cells (Figure 6F,G), suggesting species‐specific differences not only in their numbers but also in their functions. Thus, C12 may not be suitable for studies intended to apply findings from mice to humans. Cluster 10, a subpopulation exclusively detected in mouse oral mucosa, specifically expresses mouse‐specific secretoglobin gene family members (SCGBs), including SCGB1B3, SCGB1B10, SCGB1B12, SCGB1B20, SCGB2B20, SCGB2B12, SCGB2B17 and SCGB2B18 [62, 63] (Figure 6H). These genes are associated with secretory glands, including salivary glands. The ‘Abp’ genes within the Scgb1b/Scgb2b subset of the SCGB gene family have independently evolved in various mammalian lineages, representing an ‘evolutionary bloom’. In many species, including primates, this expansion has primarily resulted in pseudogenes, while in others, such as shrews and elephants, these genes have been completely lost [63]. This suggests that the SCGB genes in mice and humans have different evolutionary origins. Combining CellChat interaction analysis and GO functional enrichment analysis, we found that mouse C10 was related to various biosynthesis pathways (Figure 6I,J), with no human equivalent detected. Therefore, we speculate that C10 might be a group of mouse‐specific salivary gland epithelium related to the synthesis and secretion of mouse‐specific ABP proteins.

In conclusion, although mouse neural glial cells exhibit similar interactions and functions to those in humans, the significant difference in their numbers renders them unsuitable for human studies. Additionally, salivary gland‐related epithelial cells in mice differ in both number and function compared to their human counterparts. Therefore, these subpopulations may not be appropriate for studies intended to apply findings to human contexts.

## 4 Discussion

In this study, we integrated scRNA‐seq data from human and mouse oral mucosa to construct a single‐cell atlas of healthy oral mucosa. Our unbiased comparative analysis revealed a high degree of cross‐species transcriptome conservation, including similarities in classical marker expression and cell communication patterns across cell subsets. However, we also uncovered the species‐specific differences, including distinct cell populations unique to humans or mice, providing a nuanced understanding of oral mucosa biology.

Fibroblasts in human and mouse oral mucosa exhibited an exceptionally strong capacity for intercellular communication, highlighting their central role in tissue homeostasis [14, 64]. Our analysis revealed that oral fibroblasts, predominantly neural crest‐derived, possess stem cell‐like properties that facilitate rapid and scarless healing. We conducted pathway enrichment analyses on all fibroblast subpopulations (Figures S4–S10). Our results indicate that FIB C0 is enriched for ‘Metabolism of xenobiotics by cytochrome P450’ and ‘Complement and coagulation cascades’, indicating this fibroblast subpopulation likely functions in protective tissue responses to both environmental stressors and injury, contributing to both detoxification and immune regulation during tissue repair and homeostasis (Figure S4). FIB C1 and FIB C7 are enriched for ‘Vascular smooth muscle contraction’ and T cell response pathways, suggesting a perivascular fibroblast identity (Figures S5 and S10). FIB C2 is enriched for ‘ECM‐receptor interaction’ and ‘Focal adhesion’ pathways, indicating that this fibroblast cluster is likely involved in extracellular matrix (ECM) remodelling, structural support and cell‐matrix interactions (Figure S6). FIB C4 and FIB C5 show enrichment in the ‘TNF signaling pathway’ and ‘Cytokine‐cytokine receptor interaction’, suggesting they represent an inflammatory fibroblast population (Figures S7 and S8). FIB C6 is enriched for ‘Antigen processing and presentation’ and ‘Leukocyte transendothelial migration’, indicating a potential role in immune modulation (Figure S9). The identification of conserved fibroblast population and their molecular signatures has significant implications for wound healing therapies and regenerative medicine. Given their central role in tissue repair, fibroblasts represent potential therapeutic targets for enhancing wound healing and promoting tissue regeneration. Strategies such as fibroblast‐targeted drug delivery, growth factor modulation and cell‐based therapies could leverage the conserved regenerative properties of these cells. Furthermore, given their similarity between humans and mice, mouse models remain valuable for preclinical testing of fibroblast‐based interventions. Potential clinical applications include the treatment of chronic wounds or oral mucosal diseases such as oral lichen planus or recurrent aphthous stomatitis and the development of biomaterials that mimic the oral mucosal microenvironments. However, our findings also highlight the need for caution when translating findings from mouse models to humans, particularly for species‐specific cell types. These differences suggest that certain cell populations may not be directly comparable between species, limiting the translational relevance of mouse models for studying these specific cell types and emphasise the importance of carefully selecting appropriate models based on the specific cell types and biological processes under investigation.

To determine whether fibroblast conservation in the oral mucosa is tissue‐specific or broadly conserved, we compared it with a non‐oral mucosa dataset, such as skin. Our analysis revealed that the cellular composition overlap between human and mouse skin was significantly lower than that of the oral mucosa (Figure S11A,B). Furthermore, fibroblast subsets in the oral mucosa exhibited a higher degree of cross‐species similarity compared to those in the skin, indicating stronger evolutionary conservation (Figure S11C–F). These findings suggest that, compared to the oral mucosa, the skin displays markedly lower evolutionary conservation between humans and mice, highlighting the tissue‐specific nature of fibroblast conservation in the oral mucosa.

Our study highlights the translational potential of fibroblast conservation; the evolutionary pressures that maintain these pathways remain underexplored. One possible explanation is the need for rapid wound healing in the oral cavity, which is constantly exposed to mechanical and microbial insults. The neural crest origin of oral mucosal fibroblasts may contribute to their enhanced plasticity and regenerative capacity compared to their mesoderm‐derived counterparts in the skin. Evolutionary selection may have preserved fibroblast‐mediated repair mechanisms to maintain oral tissue integrity, which warrants further investigation. Comparative analysis with additional non‐oral mucosal tissues, such as intestinal mucosa or skin, could help elucidate whether these fibroblast properties are unique to the oral mucosa or part of a broader conserved mechanism.

While our study provides key insights into the conservation and divergence of oral mucosal cell types, several limitations should be acknowledged. First, the sample size for both human and mouse datasets was relatively small, which may limit the generalisability of our findings. Second, dataset integration biases could have influenced the identification of conserved and species‐specific cell populations. Third, our study lacks functional validation of the identified cell types and molecular pathways. Future studies should address these limitations by incorporating larger and more diverse datasets, employing advanced computational integration methods to minimise biases, and performing functional experiments to validate key findings. Additionally, alternative experimental approaches, such as in vitro co‐culture systems and organoid models, could provide complementary insights into cell–cell interactions and species‐specific differences. Exploration of the molecular mechanisms underlying fibroblast‐mediated wound healing and the functional roles of species‐specific cell types will enhance our understanding of oral mucosal biology and its translational potential.

## 5 Conclusion

While mouse models provide valuable insights into fibroblast behaviour and tissue repair, notable species‐specific differences must be considered when translating findings to human biology. Differences in fibroblast subpopulation composition, activation thresholds, immune interactions and extracellular matrix remodelling capacities can lead to divergent healing outcomes across species. For instance, certain fibroblast subsets enriched in mouse skin may be absent or functionally distinct in humans, potentially limiting the predictive value of murine data for clinical applications. To address this, future studies should integrate humanised mouse models, primary human cell‐based co‐culture systems, and advanced 3D organoid platforms. These approaches can more accurately recapitulate human tissue microenvironments, allowing for a nuanced understanding of fibroblast‐driven regeneration. Ultimately, bridging interspecies differences through multi‐model strategies will enhance the translational relevance of preclinical discoveries and inform the development of effective, patient‐specific regenerative therapies.

## Author Contributions

**Huanyu Luo:** data curation (equal), formal analysis (equal), validation (equal), writing – original draft (equal), writing – review and editing (equal). **Xiaoyi Yu:** data curation (equal), formal analysis (equal), writing – review and editing (equal). **Hongchen Sun:** writing – review and editing (equal). **Zhengwen An:** conceptualization (equal), formal analysis (equal), funding acquisition (lead), project administration (lead), resources (equal), supervision (lead), visualization (equal), writing – review and editing (lead).

## Conflicts of Interest

The authors declare no conflicts of interest.

## Supporting information


**Figures S1–S11:** jcmm70768‐sup‐0001‐Figures.pdf.

## Data Availability

Online repositories contain the datasets mentioned in this study. Data can be obtained from the corresponding author based on the reasonable request.

## References

[1] G. C. Gurtner , S. Werner , Y. Barrandon , and M. T. Longaker , “Wound Repair and Regeneration,” Nature 453, no. 7193 (2008): 314–321. 18480812 10.1038/nature07039

[2] D. Pereira and I. Sequeira , “A Scarless Healing Tale: Comparing Homeostasis and Wound Healing of Oral Mucosa With Skin and Oesophagus,” Frontiers in Cell and Development Biology 9 (2021): 682143. 10.3389/fcell.2021.682143 PMC8350526 34381771

[3] A. M. Overmiller , A. P. Sawaya , E. D. Hope , and M. I. Morasso , “Intrinsic Networks Regulating Tissue Repair: Comparative Studies of Oral and Skin Wound Healing,” Cold Spring Harbor Perspectives in Biology 14, no. 11 (2022): a041244. 36041785 10.1101/cshperspect.a041244 PMC9620853

[4] R. Iglesias‐Bartolome , A. Uchiyama , A. A. Molinolo , et al., “Transcriptional Signature Primes Human Oral Mucosa for Rapid Wound Healing,” Science Translational Medicine 10, no. 451 (2018): eaap8798. 30045979 10.1126/scitranslmed.aap8798 PMC6598699

[5] J. E. Glim , M. van Egmond , F. B. Niessen , V. Everts , and R. H. Beelen , “Detrimental Dermal Wound Healing: What Can We Learn From the Oral Mucosa?,” Wound Repair and Regeneration 21, no. 5 (2013): 648–660. 23927738 10.1111/wrr.12072

[6] A. Malassine , J. L. Frendo , and D. Evain‐Brion , “A Comparison of Placental Development and Endocrine Functions Between the Human and Mouse Model,” Human Reproduction Update 9, no. 6 (2003): 531–539. 14714590 10.1093/humupd/dmg043

[7] J. Han , A. Gallerand , E. C. Erlich , et al., “Human Serous Cavity Macrophages and Dendritic Cells Possess Counterparts in the Mouse With a Distinct Distribution Between Species,” Nature Immunology 25, no. 1 (2024): 155–165. 38102487 10.1038/s41590-023-01688-7 PMC10990619

[8] A. Laparra , S. Tricot , M. Le Van , et al., “The Frequencies of Immunosuppressive Cells in Adipose Tissue Differ in Human, Non‐Human Primate, and Mouse Models,” Frontiers in Immunology 10 (2019): 117. 30804937 10.3389/fimmu.2019.00117 PMC6371887

[9] P. B. Esparza‐Molto , C. Nuevo‐Tapioles , M. Chamorro , et al., “Tissue‐Specific Expression and Post‐Transcriptional Regulation of the ATPase Inhibitory Factor 1 (IF1) in Human and Mouse Tissues,” FASEB Journal 33, no. 2 (2019): 1836–1851. 30204502 10.1096/fj.201800756R

[10] D. Santesmasses , M. Mariotti , and V. N. Gladyshev , “Tolerance to Selenoprotein Loss Differs Between Human and Mouse,” Molecular Biology and Evolution 37, no. 2 (2020): 341–354. 31560400 10.1093/molbev/msz218 PMC6993852

[11] H. D. Zomer and A. G. Trentin , “Skin Wound Healing in Humans and Mice: Challenges in Translational Research,” Journal of Dermatological Science 90, no. 1 (2018): 3–12. 29289417 10.1016/j.jdermsci.2017.12.009

[12] D. W. Williams , T. Greenwell‐Wild , L. Brenchley , et al., “Human Oral Mucosa Cell Atlas Reveals a Stromal‐Neutrophil Axis Regulating Tissue Immunity,” Cell 184, no. 15 (2021): 4090–4104.e15. 34129837 10.1016/j.cell.2021.05.013 PMC8359928

[13] Y. Liu , T. Xu , W. Jiang , et al., “Single‐Cell Analyses of the Oral Mucosa Reveal Immune Cell Signatures,” Journal of Dental Research 102, no. 5 (2023): 514–524. 36782103 10.1177/00220345221145903

[14] K. I. Ko , B. P. DerGarabedian , Z. Chen , et al., “Distinct Fibroblast Progenitor Subpopulation Expedites Regenerative Mucosal Healing by Immunomodulation,” Journal of Experimental Medicine 220, no. 3 (2023): e20221350. 36584405 10.1084/jem.20221350 PMC9827523

[15] N. Harunari , K. Q. Zhu , R. T. Armendariz , et al., “Histology of the Thick Scar on the Female, Red Duroc Pig: Final Similarities to Human Hypertrophic Scar,” Burns 32, no. 6 (2006): 669–677. 16905264 10.1016/j.burns.2006.03.015 PMC2878281

[16] K. Q. Zhu , L. H. Engrav , N. S. Gibran , et al., “The Female, Red Duroc Pig as an Animal Model of Hypertrophic Scarring and the Potential Role of the Cones of Skin,” Burns 29, no. 7 (2003): 649–664. 14556722 10.1016/s0305-4179(03)00205-5

[17] W. Meyer , R. Schwarz , and K. Neurand , “The Skin of Domestic Mammals as a Model for the Human Skin, With Special Reference to the Domestic Pig,” Current Problems in Dermatology 7 (1978): 39–52. 752456 10.1159/000401274

[18] N. J. Vardaxis , T. A. Brans , M. E. Boon , R. W. Kreis , and L. M. Marres , “Confocal Laser Scanning Microscopy of Porcine Skin: Implications for Human Wound Healing Studies,” Journal of Anatomy 190, no. Pt 4 (1997): 601–611. 9183682 10.1046/j.1469-7580.1997.19040601.x PMC1467644

[19] T. P. Sullivan , W. H. Eaglstein , S. C. Davis , and P. Mertz , “The Pig as a Model for Human Wound Healing,” Wound Repair and Regeneration 9, no. 2 (2001): 66–76. 11350644 10.1046/j.1524-475x.2001.00066.x

[20] N. Nicolaides , H. C. Fu , and G. R. Rice , “The Skin Surface Lipids of Man Compared With Those of Eighteen Species of Animals,” Journal of Investigative Dermatology 51, no. 2 (1968): 83–89. 4980329 10.1038/jid.1968.96

[21] G. M. Gray and H. J. Yardley , “Lipid Compositions of Cells Isolated From Pig, Human, and Rat Epidermis,” Journal of Lipid Research 16, no. 6 (1975): 434–440. 1194786

[22] Z. Liang , L. H. Engrav , P. Muangman , et al., “Nerve Quantification in Female Red Duroc Pig (FRDP) Scar Compared to Human Hypertrophic Scar,” Burns 30, no. 1 (2004): 57–64. 14693087 10.1016/j.burns.2003.09.004

[23] J. Guillard and S. Schworer , “Metabolic Control of Collagen Synthesis,” Matrix Biology 133 (2024): 43–56. 39084474 10.1016/j.matbio.2024.07.003 PMC11402592

[24] T. Manon‐Jensen , N. G. Kjeld , and M. A. Karsdal , “Collagen‐Mediated Hemostasis,” Journal of Thrombosis and Haemostasis 14, no. 3 (2016): 438–448. 26749406 10.1111/jth.13249

[25] P. Ekblom , P. Lonai , and J. F. Talts , “Expression and Biological Role of Laminin‐1,” Matrix Biology 22, no. 1 (2003): 35–47. 12714040 10.1016/s0945-053x(03)00015-5

[26] B. P. Nguyen , M. C. Ryan , S. G. Gil , and W. G. Carter , “Deposition of Laminin 5 in Epidermal Wounds Regulates Integrin Signaling and Adhesion,” Current Opinion in Cell Biology 12, no. 5 (2000): 554–562. 10978889 10.1016/s0955-0674(00)00131-9

[27] T. Chen , P. Song , M. He , et al., “Sphingosine‐1‐Phosphate Derived From PRP‐Exos Promotes Angiogenesis in Diabetic Wound Healing via the S1PR1/AKT/FN1 Signalling Pathway,” Burns and Trauma 11 (2023): tkad003. 37251708 10.1093/burnst/tkad003 PMC10208895

[28] A. J. Zollinger and M. L. Smith , “Fibronectin, the Extracellular Glue,” Matrix Biology 60‐61 (2017): 27–37. 10.1016/j.matbio.2016.07.011 27496349

[29] K. Sylakowski , P. Hwang , A. Justin , et al., “Matricellular Protein Tenascin‐C Enhances Mesenchymal Stem Cell Angiogenic and Wound Healing Efficacy Under Ischemic Conditions,” Journal of Tissue Engineering and Regenerative Medicine 16, no. 12 (2022): 1249–1260. 36346015 10.1002/term.3367

[30] C. C. Yates , A. Nuschke , M. Rodrigues , et al., “Improved Transplanted Stem Cell Survival in a Polymer Gel Supplemented With Tenascin C Accelerates Healing and Reduces Scarring of Murine Skin Wounds,” Cell Transplantation 26, no. 1 (2017): 103–113. 27452449 10.3727/096368916X692249 PMC5253345

[31] J. C. Adams and J. Lawler , “The Thrombospondins,” Cold Spring Harbor Perspectives in Biology 3, no. 10 (2011): a009712. 21875984 10.1101/cshperspect.a009712 PMC3179333

[32] E. Y. Rim , H. Clevers , and R. Nusse , “The Wnt Pathway: From Signaling Mechanisms to Synthetic Modulators,” Annual Review of Biochemistry 91 (2022): 571–598. 10.1146/annurev-biochem-040320-103615 35303793

[33] J. L. Whyte , A. A. Smith , and J. A. Helms , “Wnt Signaling and Injury Repair,” Cold Spring Harbor Perspectives in Biology 4, no. 8 (2012): a008078. 22723493 10.1101/cshperspect.a008078 PMC3405869

[34] L. Plantier , C. Rochette‐Egly , D. Goven , et al., “Dysregulation of Elastin Expression by Fibroblasts in Pulmonary Emphysema: Role of Cellular Retinoic Acid Binding Protein 2,” Thorax 63, no. 11 (2008): 1012–1017. 18621984 10.1136/thx.2007.093302

[35] H. X. Liu , I. Ly , Y. Hu , and Y. J. Wan , “Retinoic Acid Regulates Cell Cycle Genes and Accelerates Normal Mouse Liver Regeneration,” Biochemical Pharmacology 91, no. 2 (2014): 256–265. 25087568 10.1016/j.bcp.2014.07.003 PMC4236911

[36] Q. M. Phan , S. Sinha , J. Biernaskie , and R. R. Driskell , “Single‐Cell Transcriptomic Analysis of Small and Large Wounds Reveals the Distinct Spatial Organization of Regenerative Fibroblasts,” Experimental Dermatology 30, no. 1 (2021): 92–101. 33237598 10.1111/exd.14244 PMC7839523

[37] S. S. Lim , S. H. Kook , and J. C. Lee , “COMP‐Ang1 Enhances DNA Synthesis and Cell Cycle Progression in Human Periodontal Ligament Cells via Tie2‐Mediated Phosphorylation of PI3K/Akt and MAPKs,” Molecular and Cellular Biochemistry 416, no. 1–2 (2016): 157–168. 27107990 10.1007/s11010-016-2704-3

[38] J. Mao , L. Chen , S. Qian , et al., “Transcriptome Network Analysis of Inflammation and Fibrosis in Keloids,” Journal of Dermatological Science 113, no. 2 (2024): 62–73. 38242738 10.1016/j.jdermsci.2023.12.007

[39] T. Tabib , C. Morse , T. Wang , W. Chen , and R. Lafyatis , “SFRP2/DPP4 and FMO1/LSP1 Define Major Fibroblast Populations in Human Skin,” Journal of Investigative Dermatology 138, no. 4 (2018): 802–810. 29080679 10.1016/j.jid.2017.09.045 PMC7444611

[40] N. Angelov , N. Moutsopoulos , M. J. Jeong , S. Nares , G. Ashcroft , and S. M. Wahl , “Aberrant Mucosal Wound Repair in the Absence of Secretory Leukocyte Protease Inhibitor,” Thrombosis and Haemostasis 92, no. 2 (2004): 288–297. 15269824 10.1160/TH03-07-0446

[41] M. Arostegui , R. W. Scott , K. Bose , and T. M. Underhill , “Cellular Taxonomy of Hic1(+) Mesenchymal Progenitor Derivatives in the Limb: From Embryo to Adult,” Nature Communications 13, no. 1 (2022): 4989. 10.1038/s41467-022-32695-1 PMC9411605 36008423

[42] A. M. Kabat , A. Hackl , D. E. Sanin , et al., “Resident T(H)2 Cells Orchestrate Adipose Tissue Remodeling at a Site Adjacent to Infection,” Science Immunology 7, no. 76 (2022): eadd3263. 36240286 10.1126/sciimmunol.add3263 PMC11905186

[43] E. E. McCartney , Y. Chung , and M. B. Buechler , “Life of Pi: Exploring Functions of Pi16+ Fibroblasts,” F1000Research 13 (2024): 126. 38919948 10.12688/f1000research.143511.2 PMC11196929

[44] L. Cai , M. G. Kolonin , and D. Anastassiou , “The Fibro‐Adipogenic Progenitor APOD+DCN+LUM+ Cell Population in Aggressive Carcinomas,” Cancer Metastasis Reviews 43, no. 3 (2024): 977–980. 38466528 10.1007/s10555-024-10181-y PMC11300568

[45] Q. Li , Z. Zhu , L. Wang , et al., “Single‐Cell Transcriptome Profiling Reveals Vascular Endothelial Cell Heterogeneity in Human Skin,” Theranostics 11, no. 13 (2021): 6461–6476. 33995668 10.7150/thno.54917 PMC8120211

[46] A. Thiriot , C. Perdomo , G. Cheng , et al., “Differential DARC/ACKR1 Expression Distinguishes Venular From Non‐Venular Endothelial Cells in Murine Tissues,” BMC Biology 15, no. 1 (2017): 45. 28526034 10.1186/s12915-017-0381-7 PMC5438556

[47] J. C. Schupp , T. S. Adams , C. Cosme, Jr. , et al., “Integrated Single‐Cell Atlas of Endothelial Cells of the Human Lung,” Circulation 144, no. 4 (2021): 286–302. 34030460 10.1161/CIRCULATIONAHA.120.052318 PMC8300155

[48] X. Guo , N. Khosraviani , S. Raju , et al., “Endothelial ACKR1 Is Induced by Neutrophil Contact and Down‐Regulated by Secretion in Extracellular Vesicles,” Frontiers in Immunology 14 (2023): 1181016. 37153544 10.3389/fimmu.2023.1181016 PMC10160463

[49] K. Ley , C. Laudanna , M. I. Cybulsky , and S. Nourshargh , “Getting to the Site of Inflammation: The Leukocyte Adhesion Cascade Updated,” Nature Reviews. Immunology 7, no. 9 (2007): 678–689. 10.1038/nri2156 17717539

[50] R. Wakasugi , K. Suzuki , and T. Kaneko‐Kawano , “Molecular Mechanisms Regulating Vascular Endothelial Permeability,” International Journal of Molecular Sciences 25, no. 12 (2024): 6415. 38928121 10.3390/ijms25126415 PMC11203514

[51] L. Rochin , I. Hurbain , L. Serneels , et al., “BACE2 Processes PMEL to Form the Melanosome Amyloid Matrix in Pigment Cells,” Proceedings of the National Academy of Sciences of the United States of America 110, no. 26 (2013): 10658–10663. 23754390 10.1073/pnas.1220748110 PMC3696817

[52] Y. Xi , H. Liu , L. Li , et al., “Transcriptome Reveals Multi Pigmentation Genes Affecting Dorsoventral Pattern in Avian Body,” Frontiers in Cell and Development Biology 8 (2020): 560766. 10.3389/fcell.2020.560766 PMC7559526 33117797

[53] R. J. B. Cordero and A. Casadevall , “Melanin,” Current Biology 30, no. 4 (2020): R142–R143. 32097632 10.1016/j.cub.2019.12.042

[54] J. P. Ortonne , “Photoprotective Properties of Skin Melanin,” British Journal of Dermatology 146, no. Suppl 61 (2002): 7–10. 10.1046/j.1365-2133.146.s61.3.x 11966725

[55] T. Santana , A. Queiroz , L. M. C. Goncales , N. S. Andrade , and M. Trierveiler , “Focal Melanocytic Lesions of the Oral Mucosa: An Epidemiological and Morphological Study,” Oral Diseases 29, no. 7 (2023): 2723–2733. 36565435 10.1111/odi.14482

[56] A. Noble , R. Qubrosi , S. Cariba , K. Favaro , and S. L. Payne , “Neural Dependency in Wound Healing and Regeneration,” Developmental Dynamics 253, no. 2 (2024): 181–203. 37638700 10.1002/dvdy.650

[57] S. Suvas , “Role of Substance P Neuropeptide in Inflammation, Wound Healing, and Tissue Homeostasis,” Journal of Immunology 199, no. 5 (2017): 1543–1552. 10.4049/jimmunol.1601751 PMC5657331 28827386

[58] B. Laverdet , A. Danigo , D. Girard , L. Magy , C. Demiot , and A. Desmouliere , “Skin Innervation: Important Roles During Normal and Pathological Cutaneous Repair,” Histology and Histopathology 30, no. 8 (2015): 875–892. 25799052 10.14670/HH-11-610

[59] D. J. Culp , L. R. Latchney , M. A. Fallon , et al., “The Gene Encoding Mouse Muc19: cDNA, Genomic Organization and Relationship to Smgc,” Physiological Genomics 19, no. 3 (2004): 303–318. 15340121 10.1152/physiolgenomics.00161.2004

[60] Y. Chen , Y. H. Zhao , T. B. Kalaslavadi , et al., “Genome‐Wide Search and Identification of a Novel Gel‐Forming Mucin MUC19/Muc19 in Glandular Tissues,” American Journal of Respiratory Cell and Molecular Biology 30, no. 2 (2004): 155–165. 12882755 10.1165/rcmb.2003-0103OC

[61] L. Innocentini , A. A. Silva , M. A. Carvalho , et al., “Salivary BPIFA Proteins Are Altered in Patients Undergoing Hematopoietic Cell Transplantation,” Oral Diseases 28, no. 4 (2022): 1279–1288. 33682222 10.1111/odi.13832

[62] B. C. Jackson , D. C. Thompson , M. W. Wright , et al., “Update of the Human Secretoglobin (SCGB) Gene Superfamily and an Example of ‘evolutionary Bloom’ of Androgen‐Binding Protein Genes Within the Mouse Scgb Gene Superfamily,” Human Genomics 5, no. 6 (2011): 691–702. 22155607 10.1186/1479-7364-5-6-691 PMC3251818

[63] C. M. Laukaitis , A. Heger , T. D. Blakley , P. Munclinger , C. P. Ponting , and R. C. Karn , “Rapid Bursts of Androgen‐Binding Protein (Abp) Gene Duplication Occurred Independently in Diverse Mammals,” BMC Evolutionary Biology 8 (2008): 46. 18269759 10.1186/1471-2148-8-46 PMC2291036

[64] X. Xu , C. Chen , K. Akiyama , et al., “Gingivae Contain Neural‐Crest‐ and Mesoderm‐Derived Mesenchymal Stem Cells,” Journal of Dental Research 92, no. 9 (2013): 825–832. 23867762 10.1177/0022034513497961 PMC3744273

