# Supplementary material for: Delineating Tissue‐Specific Cell Identity of Oral Mucosa in Humans and Mice From a Single‐Cell Perspective
Source: J Cell Mol Med. 2025 Aug 1;29(15):e70768. doi: 10.1111/jcmm.70768 (PMC12316598; doi:10.1111/jcmm.70768)

SFig. 1 UMAP showing pre- and post-correction integration.

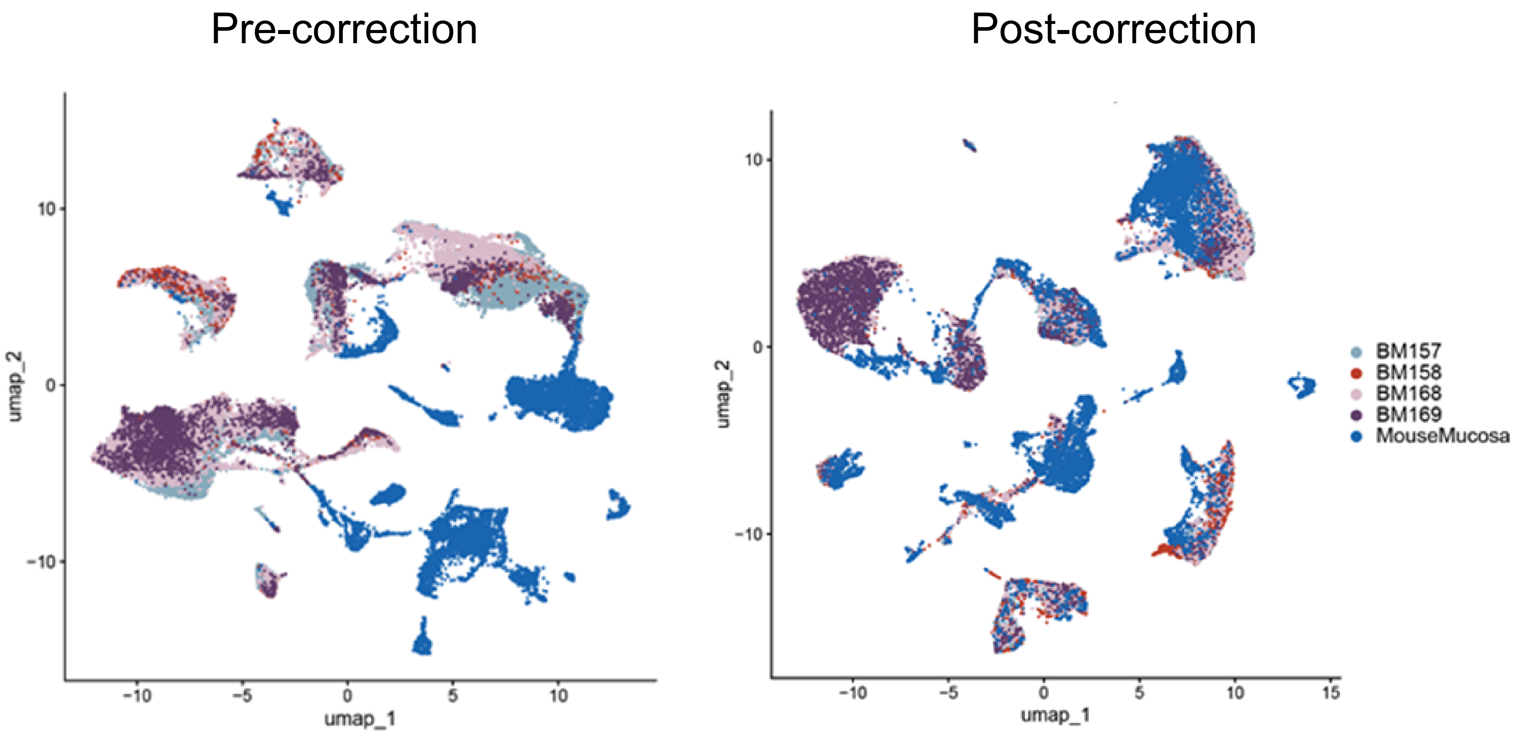

SFig. 2 CellChat analysis on communication strength between different cell subsets in human and mouse oral mucosa.

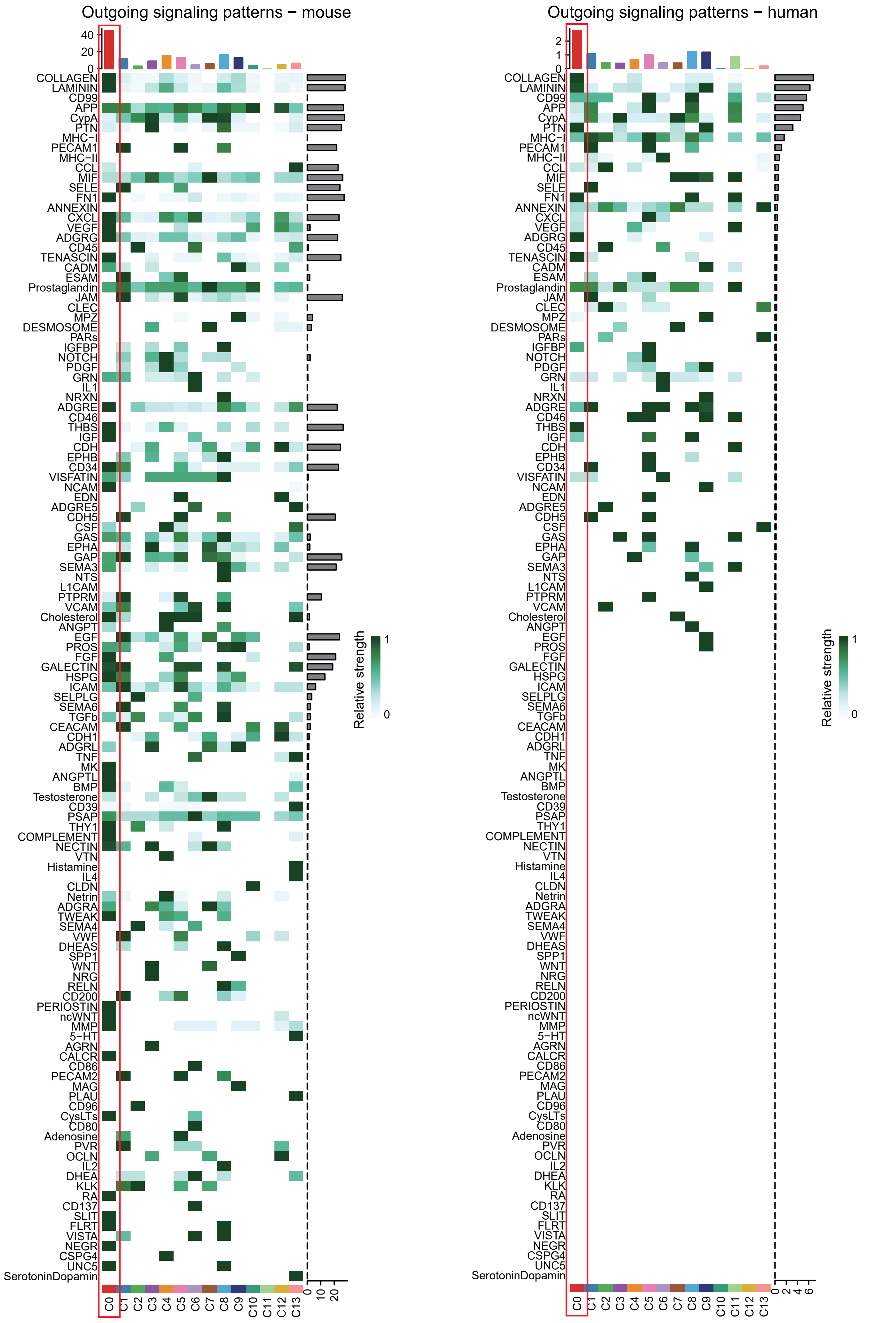

SFig. 3 Comparison of tissue-specific clusters within their respective tissue populations and across species.

A

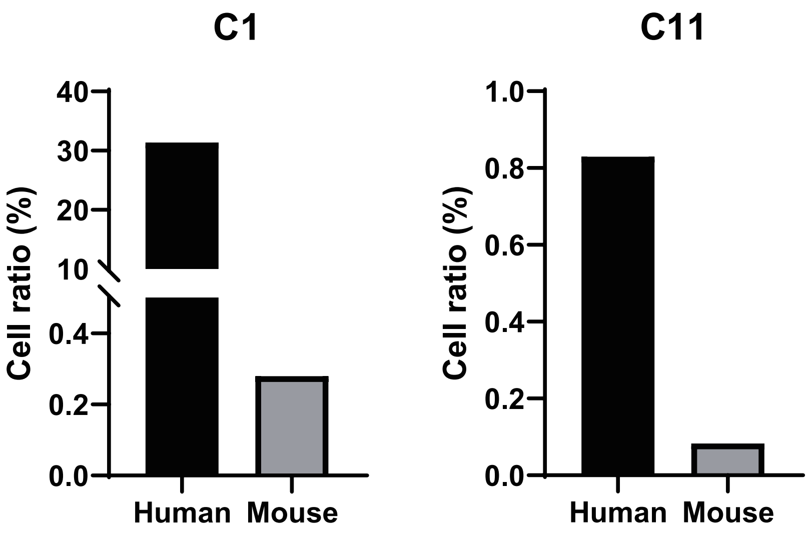

B

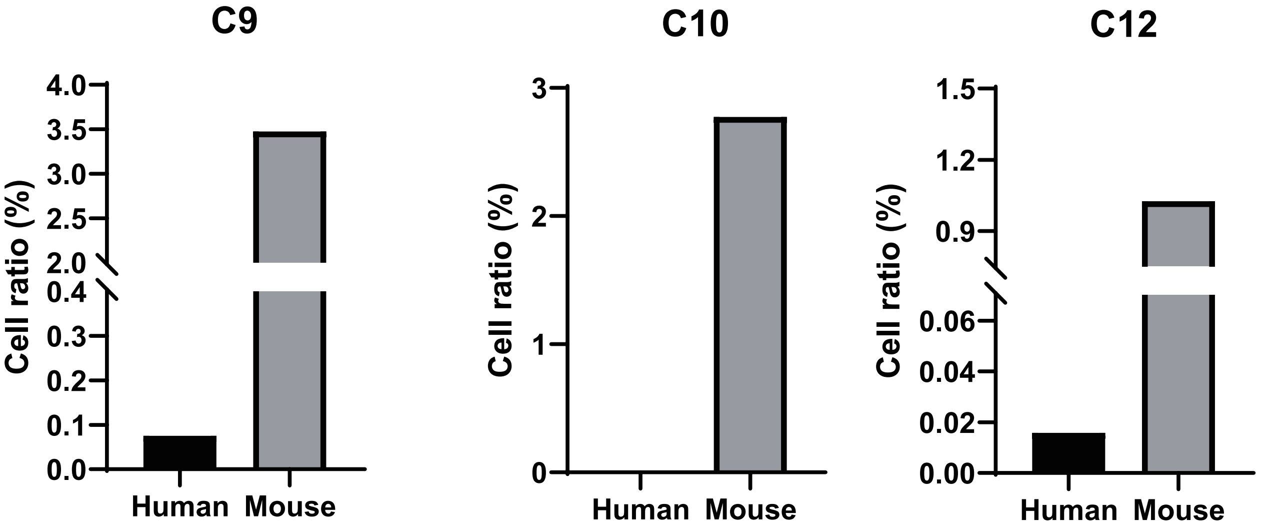

SFig. 4 Pathways enrichment analysis of mouse and human FIB C0.

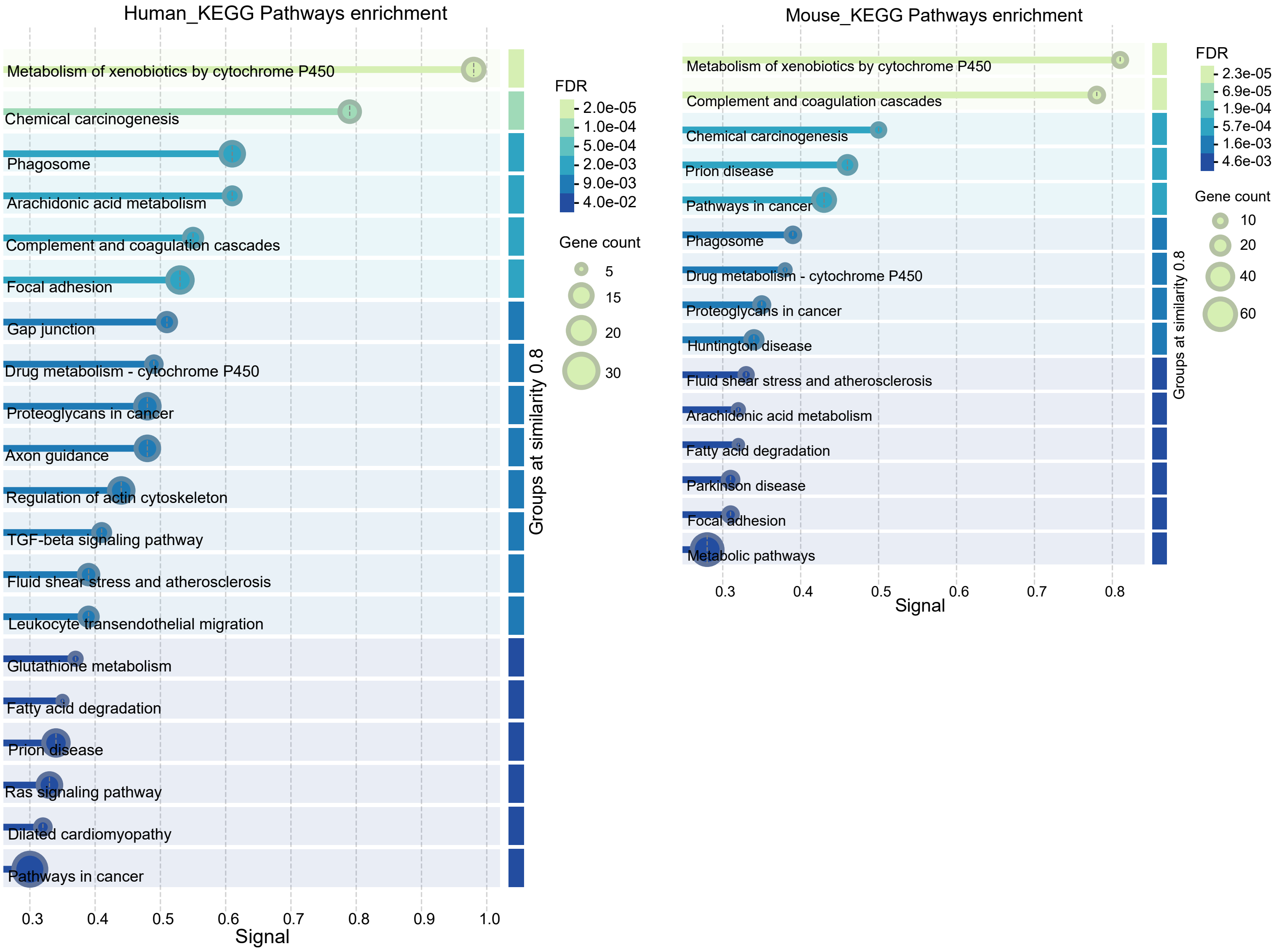

SFig. 5 Pathways enrichment analysis of mouse and human FIB C1.

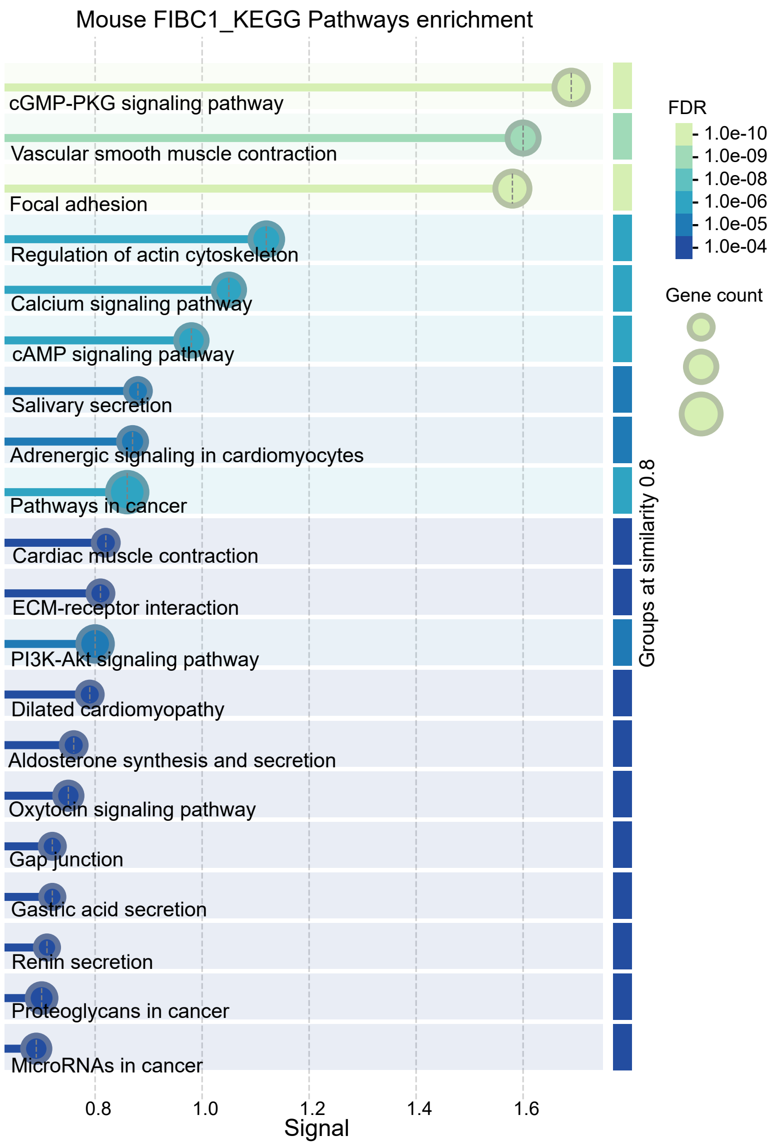

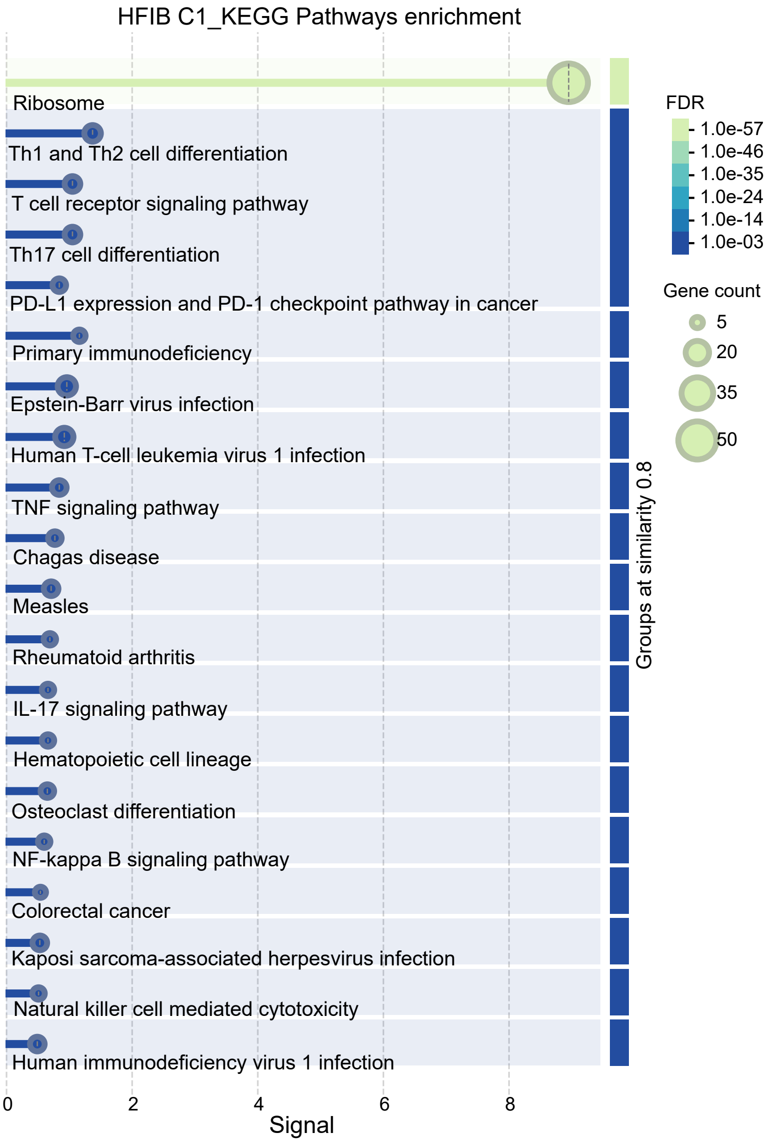

SFig. 6 Pathways enrichment analysis of mouse and human FIB C2.

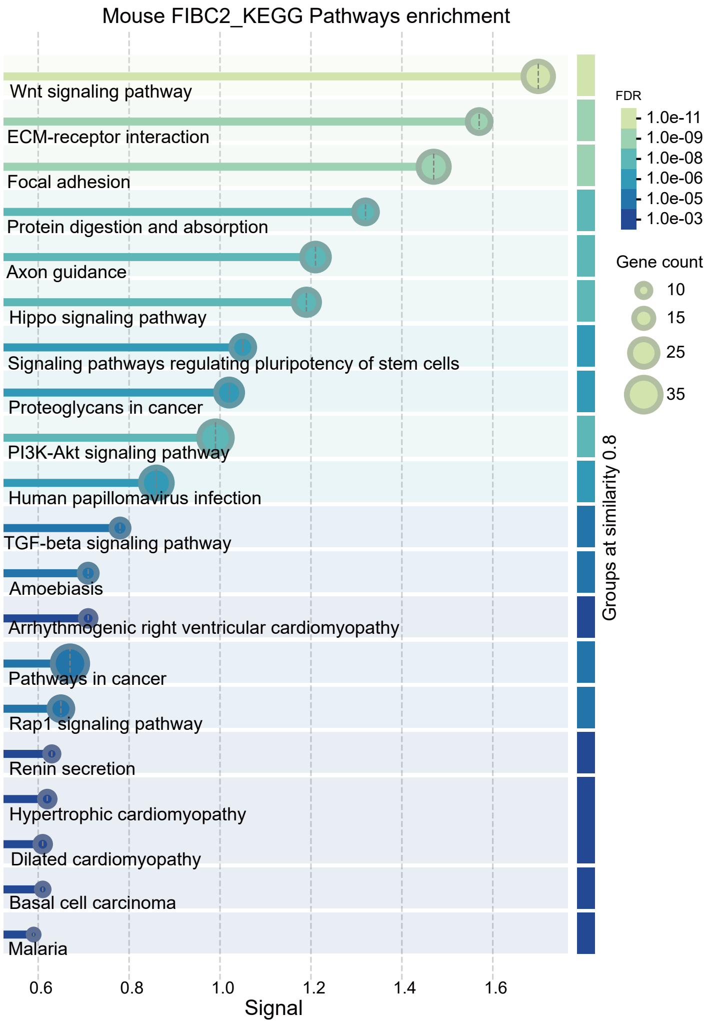

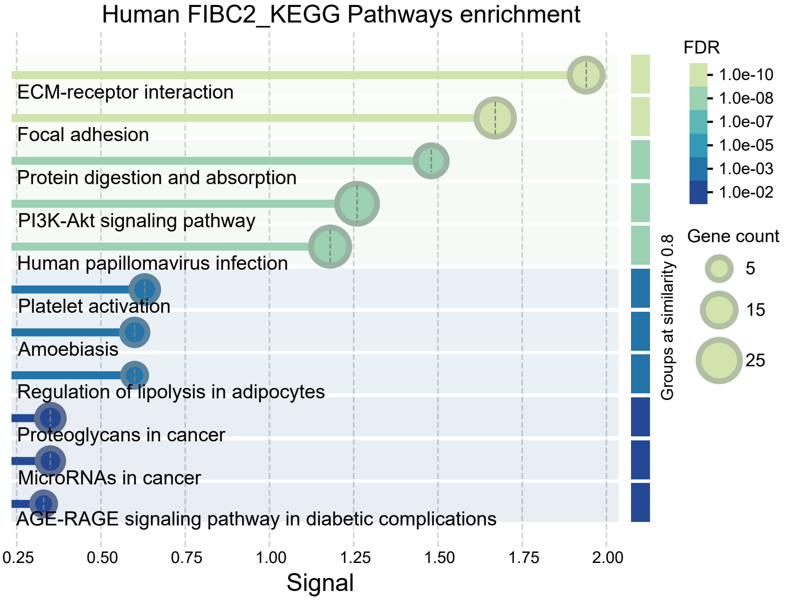

SFig. 7 Pathways enrichment analysis of mouse and human FIB C4.

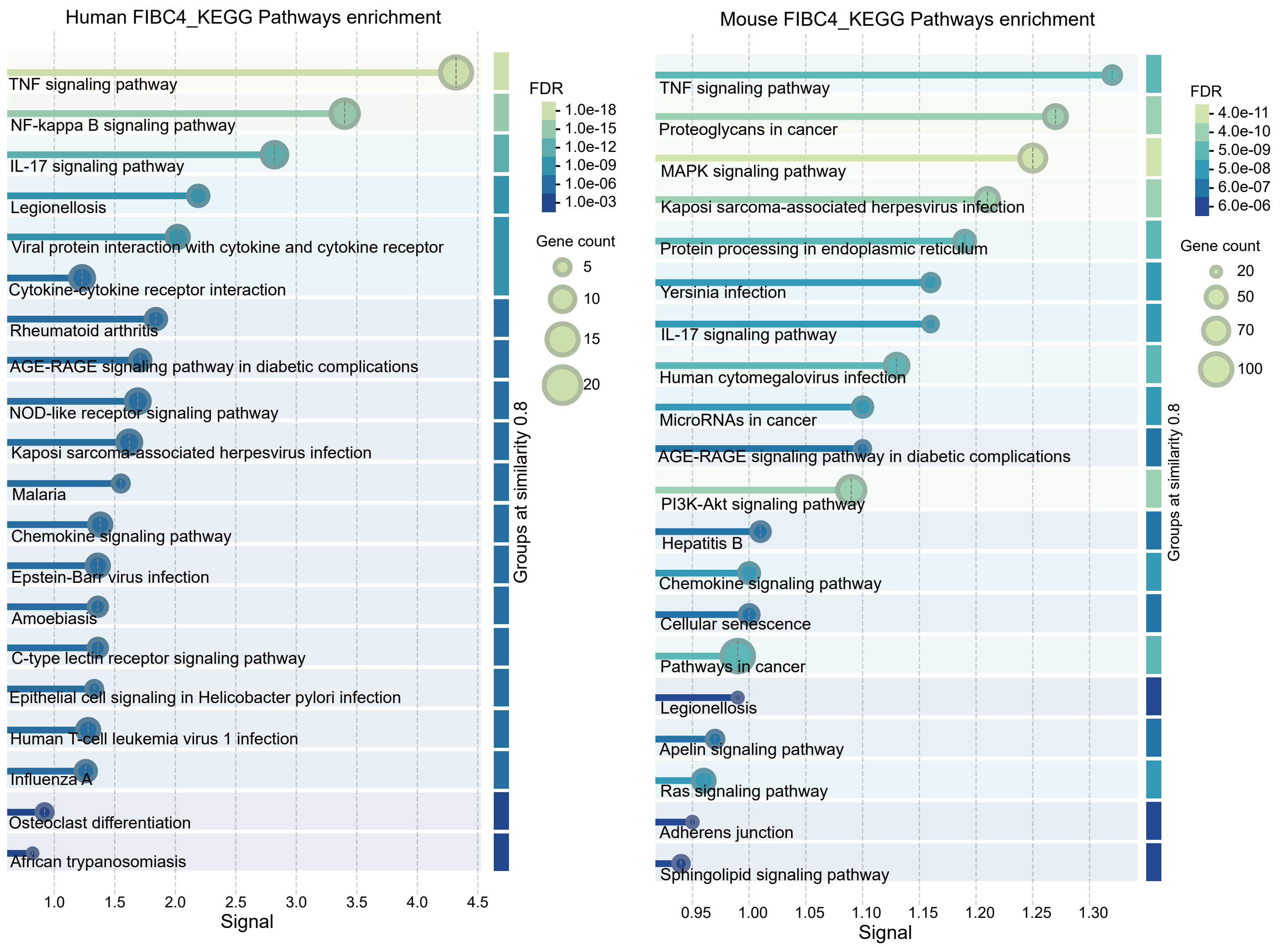

SFig. 8 Pathways enrichment analysis of mouse and human FIB C5.

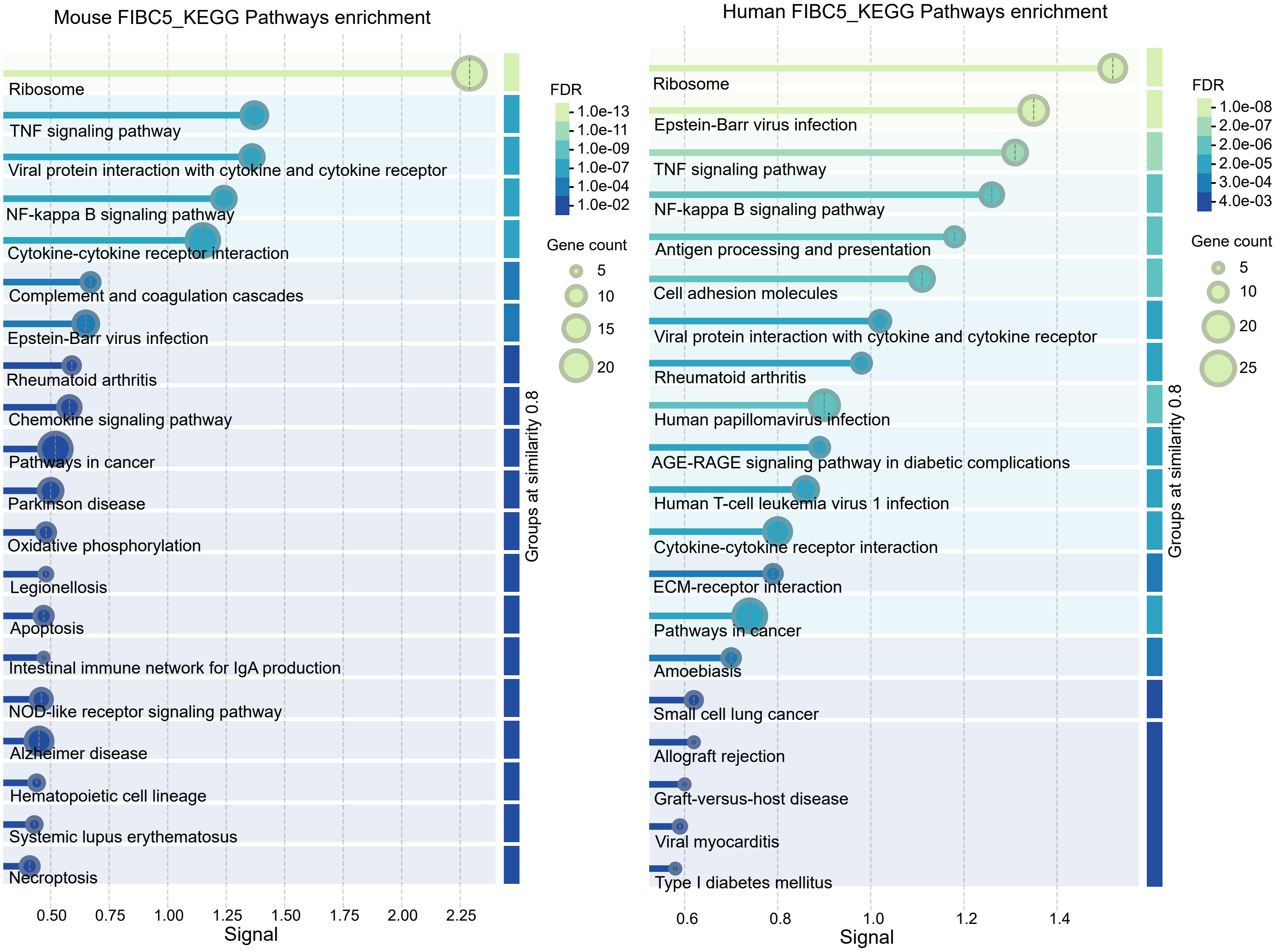

SFig. 9 Pathways enrichment analysis of mouse and human FIB C6.

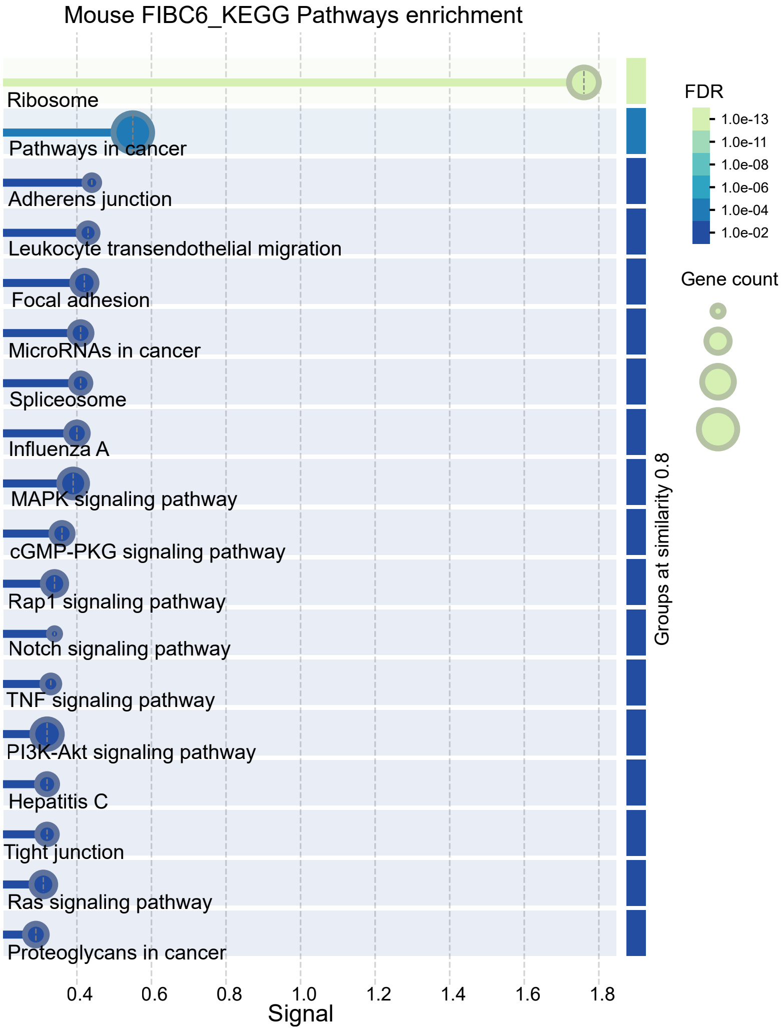

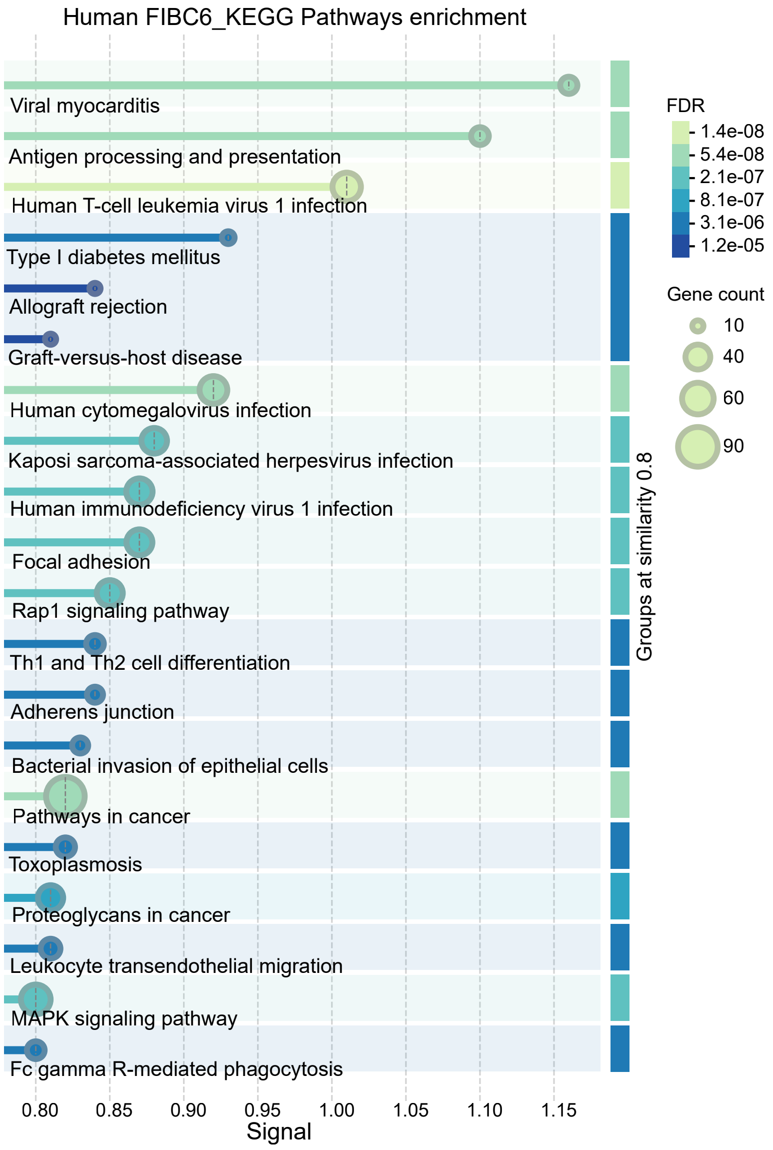

SFig. 10 Pathways enrichment analysis of mouse and human FIB C7.

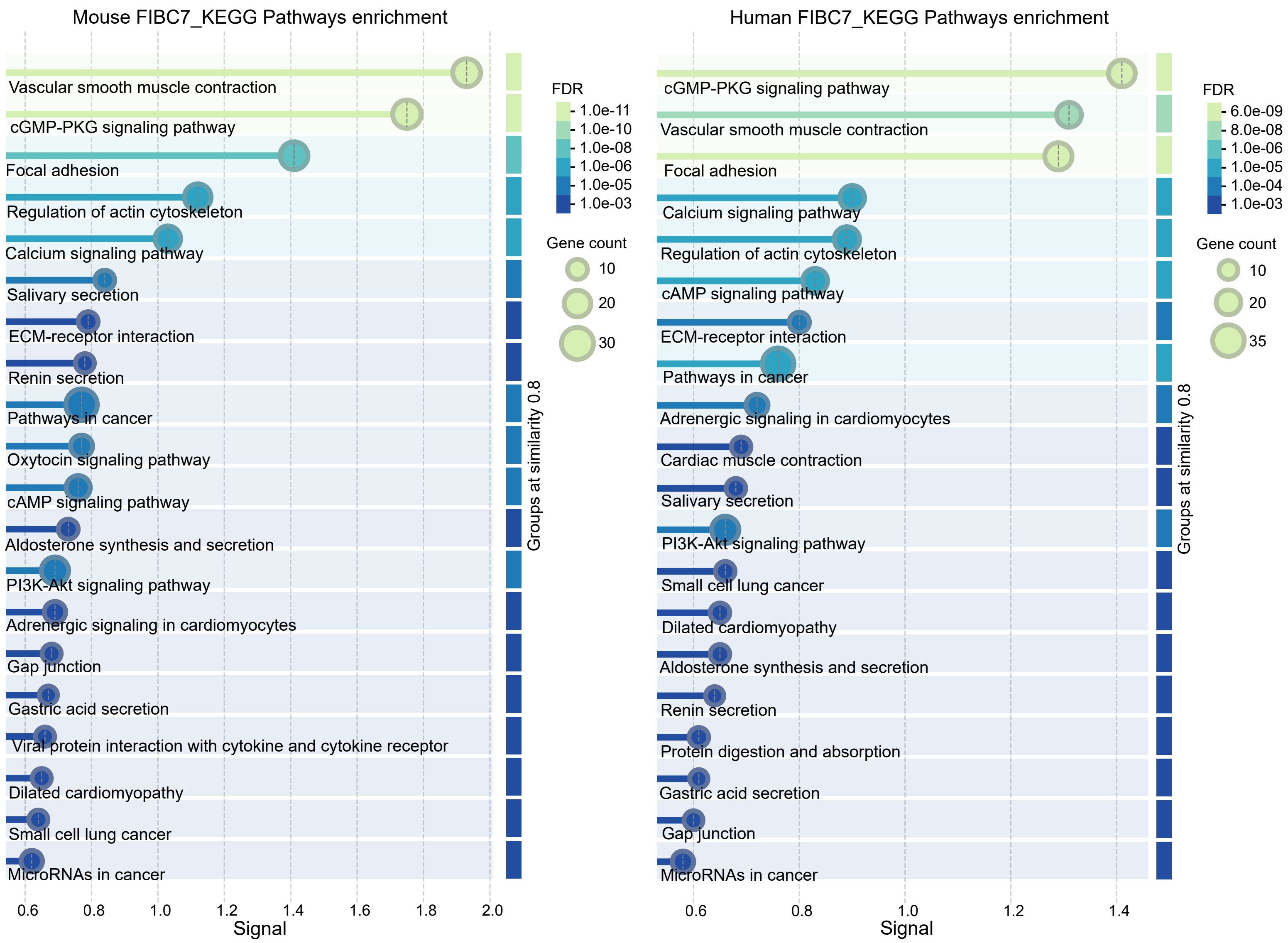

SFig. 11 The cross-species comparative analysis of skin between humans and mice.

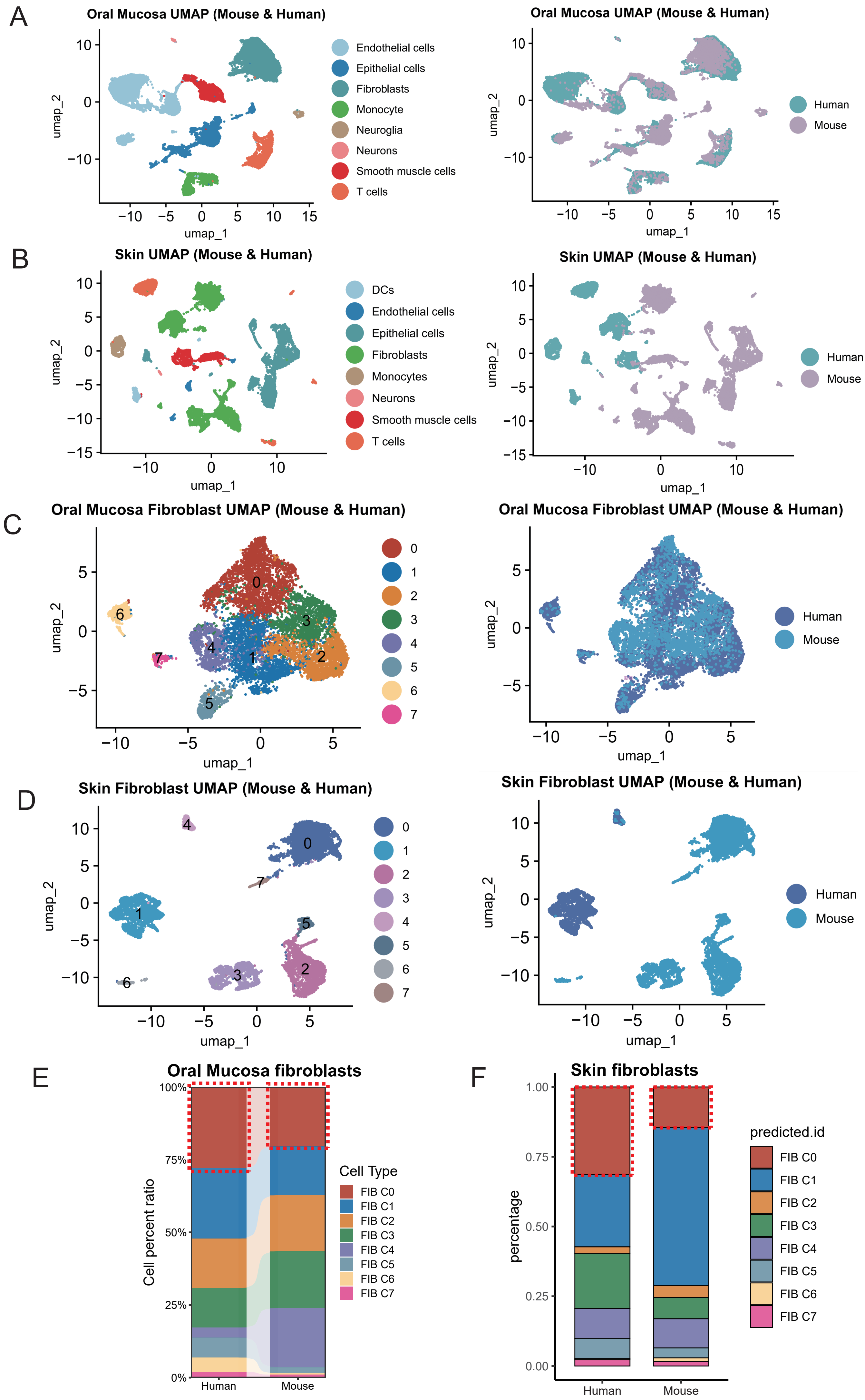

Supplement: Supplementary file 1 — Figures S1–S11: jcmm70768‐sup‐0001‐Figures.pdf. [file JCMM-29-e70768-s001.pdf]
